# Genomic, Evolutionary and Phenotypic Insights into *Pseudomonas* Phage Adele, a Novel *Pakpunavirus* with Potential for Phage Therapy

**DOI:** 10.3390/v18010042

**Published:** 2025-12-25

**Authors:** Andrei V. Chaplin, George A. Skvortsov, Nina N. Sykilinda, Konstantin S. Troshin, Anna A. Vasilyeva, Artem A. Malkov, Maria R. Leont’eva, Konstantin A. Miroshnikov, Mikhail A. Yaitsky, Dmitriy A. Shagin, Boris A. Efimov, Lyudmila I. Kafarskaia, Sergei K. Komarevtsev, Peter V. Evseev

**Affiliations:** 1 Pirogov Russian National Research Medical University, Ostrovityanova Str., 1, 117997 Moscow, Russia; galaxy22242500@mail.ru (G.A.S.); konstantinetr@gmail.com (K.S.T.); annavasilyeva.bioscience@gmail.com (A.A.V.); xkairyx@gmail.com (A.A.M.); x_blade@inbox.ru (M.R.L.); yaitskiyma@gmail.com (M.A.Y.); shagdim777@gmail.com (D.A.S.); efimov_ba@mail.ru (B.A.E.); likmed@mail.ru (L.I.K.); 2 Shemyakin-Ovchinnikov Institute of Bioorganic Chemistry, Russian Academy of Sciences, Miklukho-Maklaya Str. 16/10, 117997 Moscow, Russia; sykilinda@mail.ru (N.N.S.); kmi@bk.ru (K.A.M.); skomarevtsev@yandex.ru (S.K.K.)

**Keywords:** phage, phage evolution, *Pakpunavirus*, *Pseudomonas aeruginosa*, *Pseudomonas* phages, phage genomics, phage therapy, antimicrobial resistance

## Abstract

Bacteriophages are powerful drivers of microbial evolution and are increasingly explored as alternatives to antibiotics against multidrug-resistant pathogens such as *Pseudomonas aeruginosa*. Here, we describe the isolation, phenotypic characterization and genomic, structural and evolutionary analysis of *Pseudomonas* phage Adele, a lytic myovirus representing a novel species within the genus *Pakpunavirus* (family *Vandenendeviridae*). Phage Adele exhibits a short latent period of 20 min, a burst size of 59 ± 11 virions per infected cell and a high virulence index, efficiently lysing non-O11 *Pseudomonas aeruginosa* strains and reducing biofilm biomass. In vivo, Adele confers marked protection in a *Galleria mellonella* infection model. Phylogenetic reconstruction, synteny analysis and structural modeling demonstrate the relatedness of *Vandenendeviridae* to phages of the *Andersonviridae* and *Vequintavirinae* clades, pointing to a stable, ancestral virion architecture that has undergone lineage-specific elaborations, including the duplication and divergence of tail tube proteins. The tail assembly chaperone gene employs a conserved −1 programmed ribosomal frameshift. Phage Adele encodes an elaborate set of metabolic reprogramming and anti-defense systems, reflecting extensive horizontal gene transfer. The combination of a conserved structural architecture and mosaic genome establishes Adele as an exemplary system for studying modular evolution in phages, alongside its demonstrated therapeutic efficacy.

## 1. Introduction

Viruses are a fundamental component of the biosphere, forming a diverse virosphere that drives genetic innovation across all cellular domains [1,2]. Tailed bacteriophages are among the most abundant biological entities, exerting top-down control on microbial communities and reshaping prokaryotic populations through infection, lysis and lysogeny [3]. Genomically, phages exemplify modular evolution: their genomes consist of functional modules that can be recombined between related viruses, producing the mosaic architecture typical of many phage genomes [4]. This modularity extends to individual protein domains, for instance in receptor-binding proteins, where N-terminal particle-binding and C-terminal receptor-recognition regions can be interchanged between distinct phages, enabling rapid shifts in host range [5]. Horizontal gene transfer mediated by viruses and virus-like elements can thus be viewed as an exchange of genetic information that alters the properties of participating lineages and, over many events, reshapes the evolutionary dynamics of microbial communities [1].

The virosphere is further enriched by virus-like mobile elements such as gene transfer agents (GTAs), phage-inducible chromosomal islands and related systems, and phage satellites. GTAs are phage-like particles that package random fragments of the producer cell’s genome and mediate gene transfer at frequencies orders of magnitude higher than spontaneous mutation, thereby contributing to horizontal gene flow [6,7]. Phage-inducible chromosomal islands (PICIs) and capsid-forming PICI-like elements (cf-PICIs) are molecular parasites that hijack helper phages, repurpose their structural proteins for their own packaging and influence horizontal gene transfer, host adaptation and virulence in Gram-positive and Gram-negative bacteria [8,9]. Phage satellites, including P4-like elements, likewise depend on helper phages for structural functions and form widespread families of mobile genetic elements in bacterial genomes [10,11]. Together, canonical lytic and temperate phages, GTAs, PICIs or cf-PICIs and satellites create a dense network of genetic exchanges between viruses and cellular organisms and underscore the central role of phages and phage-derived particles in the long-term evolution of microbial genomes and the biosphere as a whole [1]. Against this evolutionary background, bacteriophages also remain highly relevant from a biomedical perspective: their ability to specifically infect and kill bacterial cells, including multidrug-resistant strains, has led to a renewed interest in phage biology and phage therapy as a complementary or alternative approach to antibiotics [12].

The relentless rise in antimicrobial resistance (AMR) poses a catastrophic threat to modern medicine. Among the most concerning multidrug-resistant pathogens is *Pseudomonas aeruginosa*, a notorious opportunist responsible for life-threatening nosocomial infections, particularly in immunocompromised patients with chronic obstructive pulmonary disease, cystic fibrosis, cancer and burns as well as undergoing mechanical ventilation [13]. As a ubiquitous environmental bacterium, it has acquired multiple efflux pumps to resist natural xenobiotics, while in clinical and antibiotic-polluted environments, constant selective pressure from antibacterials drives further evolution of resistance [13,14]. Phage therapy, the use of viruses to specifically infect and kill bacterial hosts, represents a promising treatment alternative use due to a vast natural diversity of phages shaped by endless competitive co-evolution with their hosts.

One viral genus that exemplifies this promise is *Pakpunavirus*. It was established by the International Committee on Taxonomy of Viruses (ICTV) in 2015, based on previous reports on closely related *P. aeruginosa* phage group called PAK_P1-like or PaP1-like viruses [15,16]. It represents the most extensively studied clade within a large *Pseudomonas*-infecting lytic phage family *Vandenendeviridae* [17]. Because these phages are widespread, form large plaques, and are easily propagated, the genus has rapidly expanded to include 34 species as of the ICTV 2024 Release.

Phage particles of *Pakpunavirus* exhibit a myovirus morphotype, featuring an icosahedral head and a contractile tail with attached straight tail fibers [15,16,18,19]. The fibers are the proteins responsible for determining host specificity [20]; mutational studies [21,22,23,24] and a blocking assay [25] demonstrate that the host receptor for the phage attachment is an O-specific antigen of the lipopolysaccharide. Furthermore, mutated variants of baseplate wedge protein are shown to bind *P. aeruginosa* core oligosaccharide [25]; however, the structural details of *Pakpunavirus* virions including baseplate organization remain unstudied.

*Pakpunavirus* phages are obligate lytic phages of *P. aeruginosa*, driving transcriptional reprogramming and rapidly lysing host cells after a short latency period [23,26]. In a few minutes post infection they overwhelmingly replace host transcripts with viral transcripts, hijacking the host RNA polymerase [26]. A comparative study of *P. aeruginosa* phages ranked *Pakpunavirus* members as highly suitable candidates for phage therapy due to a broad host range, high virulence index due to their rapid and effective lytic cycle, and an absence of genes for integration proteins, virulence factors or antibiotic resistance [27]. No cytotoxicity of *Pakpunaviruses* toward human cells has been previously reported [28,29]. While alveolar macrophages actively phagocytize *Pakpunaviruses* in a mouse model of acute pneumonia, neutrophils and phages act synergistically to eliminate phage-resistant subpopulations [30,31]. In human neutrophils these phages stimulate IL-8 secretion but do not activate oxidative burst pathways or induce NETosis [29]. These findings suggest that *Pakpunaviruses* may actively recruit the cellular arm of the immune system to the site of infection while avoiding excessive inflammation, a highly desirable trait for a therapeutic agent.

*Pakpunavirus* members exhibit significant variation in host range [27]. However, the genetic determinants that govern both host adsorption and evasion of bacterial defense systems remain poorly described. Furthermore, current sequencing efforts of new isolates are insufficient to assess the evolutionary conservation and functional significance of specific genomic elements. Understanding these determinants is critical for the ability to rationally select or engineer phages with predictable and broad host range. To address this gap, in this study we present a detailed analysis of phage Adele, a novel species within the genus *Pakpunavirus*, and provide its evolutionary, genomic and phenotypic characterization in the context of newly available structural and phage-host interplay data for related viruses.

## 2. Materials and Methods

### 2.1. Phage Isolation, Purification and Sequencing

*Pseudomonas aeruginosa* PAO1, obtained from the collection of Professor V.N. Krylov (I.I. Mechnikov Research Institute of Vaccines and Serums, Moscow, Russia), served as the host strain for bacteriophage propagation. *Pseudomonas* phage Adele was isolated from the water obtained from a tributary stream of Lake Baikal. Water samples (100 mL) were filtered through nitrocellulose membranes (0.45 μm pore). Five mL of *Pseudomonas aeruginosa* PAO1 overnight culture and 10 mL of 10 × Lysogeny broth (LB) (Becton-Dickinson, Franklin Lakes, NJ, USA) were added to 85 mL of filtrate, and 1 × LB was used for inoculation as a cell growth control. The enrichment and control cultures were incubated at 37 °C for 24 h in an orbital shaker. From the enrichment cultures where clarification was observed compared to the control, indicating possible lysis by bacteriophages, a 1 mL aliquot was taken and mixed with chloroform (30 µL), followed by incubation for 30 min at 4 °C. The suspension was centrifuged (10 min, 1700× *g*), and the supernatant was titrated to obtain a series of 10-fold dilutions in sterile 0.9% NaCl. From each dilution, 10 µL were dropped on double-layer LB agar plates [32] containing 10^7^ CFUs/mL of *P. aeruginosa* PAO1 in 0.75% top agar. Individual plaques were picked and propagated in double-layer agar plates two times in order to purify the phages. To propagate the bacteriophage derived from a single plaque, *P. aeruginosa* PAO1 was infected in liquid culture at 37 °C. Upon complete lysis, the bacteriophage lysate was subjected to chloroform treatment followed by centrifugation at 8000× *g* and precipitation using polyethylene glycol (10%) (NeoFroxx, Einhausen, Germany)/NaCl (1M), followed by centrifugation at 8000× *g* (Nuve NF 1200 R, NÜVE, Ankara, Turkiye) and resuspension in SM buffer (50 mM Tris-HCl, 100 mM NaCl, 8 mM MgSO4, pH 7.5).

Phage DNA was isolated from concentrated and purified high titer phage stock using phenol-chloroform method after incubation with 1% SDS and 50 µg/mL proteinase K at 65 °C for 20 min. Shearing was performed using a Bioruptor sonicator (Diagenode, Liège, Belgium). Samples underwent quality control via agarose gel electrophoresis. DNA concentration was measured on a Qubit 3.0 fluorometer (Life Technologies ThermoFisher, Waltham, MA, USA) using the Qubit dsDNA BR Assay Kit (Thermo Fisher Scientific, Waltham, MA, USA) according to the manufacturer’s protocol. Sequencing libraries were prepared from using the MGIEasy FS DNA Library Prep Set (MGI Tech Co., Shenzhen, China). After fragmentation, size selection was performed with MGIEasy DNA Clean Beads. Library concentrations were measured using the Qubit dsDNA HS Assay Kit. The final library was circularized and sequenced in paired-end mode on a DNBSEQ-G99 platform using the Universal Sequencing Reaction Kit G99 SM App-D FCU PE150 (MGI Tech Co., Shenzhen, China).

### 2.2. Transmission and Scanning Electron Microscopy

The virion structure was visualized using transmission electron microscopy. Concentrated and purified phage samples were prepared by adsorption onto grids followed by negative staining with 1% uranyl acetate (pH 4.0). Images were acquired using a LEO Omega 912 AB electron microscope (Carl Zeiss SMT, Oberkochen, Germany). The experiment was performed in two biological replicates. Phage sizes were obtained from measurements of 20 individual virions. In a separate experiment to generate virions with contracted tails, phage and bacterial preparations were mixed in a 1.5 mL microcentrifuge tube (final volume, 100 µL) and incubated for 15 min at room temperature prior to adsorption and staining with 2% uranyl acetate; samples were examined using a transmission electron microscope JEM-1011 (JEOL, Tokyo, Japan) operated at 80 kV under standard conditions.

Prior to scanning electron microscopy (SEM), *P. aeruginosa* PAO1 culture and culture mixed with phage Adele at multiplicity of infection (MOI) 100 was stored for 3 days at +4 °C. After that, suspensions in 2 mL microcentrifuge tubes were gently diluted [1:1, *v*/*v*] with phosphate-buffered saline (PBS; pH 7.4; PanReac AppliChem, Darmstadt, Germany) supplemented with 5% (*w*/*v*) sucrose to mitigate osmotic shock during partial air-drying. Aliquots (≈ [10–20] µL) were dispensed onto clean glass coverslips. After ~30 min at room temperature to allow partial drying and adhesion, samples were fixed in 2.5% glutaraldehyde (Electron Microscopy Sciences, Hatfield, PA, USA) prepared in PBS with 5% sucrose for 15 min. Coverslips were then dehydrated in graded ethanol (30%, 50%, 70%, 80%, 95% for 15 min each), followed by 100% ethanol twice for 10 min. The solvent was replaced with acetone (2 × 10 min). Samples were mounted on SEM stubs using carbon adhesive tabs and sputter-coated with a thin gold layer in argon plasma. Imaging was performed on a scanning electron microscope JSM-6380 (JEOL Ltd., Tokyo, Japan) operated at 20 kV under standard conditions.

### 2.3. Phage Adsorption, Lytic Efficiency and One-Step Growth Curve

To determine adsorption period of phage Adele, an adsorption assay was performed according to standard protocol [33]. An overnight *P. aeruginosa* PAO1 culture was added to 15 mL of LB broth in 50 mL Eppendorf tubes and cultivated 2.5 h at 37 °C with shaking at 250 rpm (ES-20 orbital shaker, Biosan, Riga, Latvia). When optical density at 600 nm (OD_600_) ~0.4 was reached, phage was added at MOI of 0.001 and tubes were vortexed. Aliquots (100 μL) were taken at time 0, 1, 2, 3, 4, 5, 10 and 15 min after phage was added, mixed with 850 μL of SM-buffer and 50 μL of chloroform (Aldosa, Moscow, Russia), vortexed and placed on ice for 15 min. Following centrifugation at 13,000× *g*, (Nuve NF 1200 R, NÜVE, Ankara, Turkiye) the supernatant was titrated to determine the number of unadsorbed phages at each time point. The adsorption curve was made by plotting the ratio of unadsorbed phage (P) to the initial phage titer (P0). The experiment was performed with three biological replicates.

The lytic efficiency of phage Adele was evaluated in a dynamic antibacterial activity assay [3]. The experiment was set up with an MOI gradient (MOI = 1, 0.1, 0.01, 10^−3^, 10^−4^, 10^−5^, 10^−6^, 10^−7^). Specifically, 100 µL of a host bacterial suspension in the logarithmic growth phase (10^7^ CFU/mL) and 10 µL of a serially diluted phage suspension (10^8^–10^1^ PFU/mL) were inoculated into a 96-well plate to achieve the predetermined MOI. A control well containing an equal volume of bacterial suspension without phage was included. The plate was incubated in a thermoshaker at 37 °C with constant shaking. The OD_630_ was measured every 30 min for 6.5 h. The reduction in absorbance relative to the control group was calculated for each MOI to characterize the phage’s lytic kinetics. Virulence index and local virulence values were calculated as described by Storms et al. [34].

To determine the latent period and burst size the one-step growth experiment was performed with three biological replicates according to [35] with modifications. Briefly, 20 mL culture of *P. aeruginosa* PAO1 at OD_600_ ~0.4 were centrifuged at 8000× *g* (Nuve NF 1200 R, NÜVE, Ankara, Turkiye), resuspended in 0.5 mL of LB, infected with 100 µL of phage at MOI of ~0.01, and incubated for 5 min. Unbound phages were removed by centrifugation for 2 min at 13,000× *g*, and the pellet was resuspended in 10 mL of LB. The suspension was incubated in 50 mL Eppendorf tubes at 37 °C on a shaker for 70 min at 180 rpm (ES-20 orbital shaker, Biosan, Riga, Latvia), with 100 µL samples taken immediately after phage was added and at 10 min intervals up to 70 min from the start of the experiment. The experiment was performed with three biological replicates. The samples were titrated and 10 µL aliquots were spotted onto double-layer agar, and plaques were counted after 24 h to determine PFU/mL. Phage burst size was calculated as the ratio of average titer at the plateau after the first burst to the total number of phages used to infect the cells at t0.

### 2.4. Testing of Antibiofilm Activity of the Phage

*P. aeruginosa* PAO1 biofilms were grown on 96-well plates (Nest Biotechnology, Wuxi, China) [36]. Each well was inoculated with 100 µL of LB broth and 100 µL of a bacterial suspension (~10^6^ CFU/mL). The plates were then incubated for 24 h at 37 °C to allow for biofilm formation. After incubation, the established biofilms were washed gently with distilled water. Then, 100 µL of the phage suspension (at various MOIs) or, for controls, 100 µL of fresh LB broth, was added to the respective wells. The plates were incubated for another 24 h at 37 °C. Following this treatment, the wells were washed again with distilled water. To stain the adherent biofilm, 100 µL of a 1% crystal violet solution (BD BBL™, Sparks, MD, USA) was added to each well, and the plate was incubated for 15 min at room temperature. The excess stain was rinsed off thoroughly under running tap water until the water became clear, and the plate was air-dried. To solubilize the dye bound to the biofilm, 200 µL of an ethanol–acetic acid–water solution (30:30:40, *v*/*v*/*v*) was added to each well. The optical density at 595 nm was then measured using a microplate spectrophotometer (Hangzhou Miu Instruments Co., Ltd., Hangzhou, China).

### 2.5. Phage Stability Under Different Conditions

Phage stability under various conditions was assessed following a modified protocol based on [37]. To determine thermal stability, a phage suspension was prepared in SM buffer at a final concentration of 7.0 × 10^8^ PFU/mL. This suspension was incubated at 30, 40, 50, 60, and 70 °C for 1 h using a TDB-120 dry block thermostat (Biosan, Riga, Latvia). To assess pH stability, the SM buffer was adjusted with NaOH or HCl to pH values of 3, 5, 7, 9, and 11. These adjusted buffers were mixed with phage samples to achieve a final titer of 7.0 × 10^6^ PFU/mL and incubated at 25 °C for 1 h. To determine UV resistance, high-titer phage samples (2.0 × 10^6^ PFU/mL) in a volume of 100 µL contained in polypropylene test tubes were exposed to a PL-S 9W/12/2p UV lamp (Philips, Amsterdam, The Netherlands) emitting at 280–315 nm. The samples were placed at a fixed distance of 20 cm vertically below the lamp, where the measured UV-C irradiance (254 nm equivalent) was 0.85 mW/cm^2^, corresponding to a fluence rate of 8.5 J/m^2^·s. The total exposure lasted 10 min. Aliquots of 50 µL were collected at 2, 5, 8, and 10 min into separate pre-chilled microcentrifuge tubes protected from light. The absorbed dose for each time point was calculated as follows: 2 min—1020 J/m^2^; 5 min—2550 J/m^2^; 8 min—4080 J/m^2^; 10 min—5100 J/m^2^. Aliquots of 50 µL were collected at 2, 5, 8, and 10 min into separate tubes. Chloroform sensitivity was evaluated by mixing phage solutions with chloroform to achieve final concentrations of 0%, 5%, 25%, 50%, and 75% (*v*/*v*), resulting in a final phage titer of 1.0 × 10^6^ PFU/mL. These mixtures were incubated at 37 °C for 30 min with shaking, according to [38]. Subsequently, the solutions were centrifuged at 10,000× *g* for 10 min, and the supernatant was collected. All resulting solutions were titrated using the double-layer agar (LB top agar) method. After 24 h of incubation, the titer in each sample was determined. All experiments were performed with three biological replicates.

### 2.6. Phage Host Range Determination

The lytic activity and host range of the bacteriophage were evaluated against a panel of 39 clinical and environmental *P. aeruginosa* isolates utilizing the double-layer agar assay. Clinical isolates with sequenced genome sequences, previously described in [39,40], were obtained from the Russian Children’s Clinical Hospital. We also tested phage Adele against other bacteria of *Pseudomonas* genus. *Pseudomonas savastanoi* pv. *glycinea* G1 (microorganisms collection of Russian State Agrarian University-Moscow Timiryazev Agriculture Academy), *Pseudomonas savastanoi* pv. *glycinea* CFBP 2214 (International Center for Microbial Resources, Collection for Plant-associated Bacteria, France) [41], *Pseudomonas protegens* CHA0 [42] and *Pseudomonas savastanoi* B-1546 (All-Russian Collection of microorganisms) were kindly provided by Dr. R.I. Tarakanov (Russian State Agrarian University-Moscow Timiryazev Agriculture Academy).

Overnight cultures of *P. aeruginosa* (200 μL) were combined with 3 mL of 0.75% soft agar and subsequently plated. Bacteriophage suspensions (∼10^9^ PFU/mL) were then spotted onto the prepared bacterial lawns and incubated at 37 °C for 18–24 h. Following incubation, the plates were carefully examined to assess bacteriophage lytic activity and host specificity. The experiment was performed in duplicate.

### 2.7. Testing Phage Activity in Vivo Using Galleria Mellonella and Zophobas Morio Models

Larvae of *Galleria mellonella* and *Zophobas morio* were obtained from a local vendor. For the infection experiments, each species was divided into three groups (*n* = 15 per group). *G. mellonella* larvae were stored upon arrival in a ventilated glass jar in the dark at room temperature and were used in experiments within 24 h. *Z. morio* larvae, which were shipped in their final instar stage, were reared on sawdust and maintained in a ventilated plastic box at room temperature. Larvae were used a week after obtaining.

The *P. aeruginosa* inoculum was prepared by first culturing the bacterium overnight in LB medium at 37 °C with shaking at 250 rpm. This overnight culture was diluted 1:100 in fresh LB medium and incubated again under the same conditions until it reached an optical density at 600 nm (OD_600_) of 0.6–0.8. The cells were then centrifuged and resuspended in saline to final concentrations of 1.0 × 10^6^ CFU/mL for *G. mellonella* and 1.0 × 10^7^ CFU/mL for *Z. morio* infections. The final infectious doses per larva were approximately 5 × 10^3^ CFU for *G. mellonella* and 1 × 10^5^ CFU for *Z. morio*.

Phage suspension concentrations for these models were 2.6 × 10^9^ PFU/mL and 2.6 × 10^10^ PFU/mL, respectively.

Larvae of *G. mellonella* were randomly selected and grabbed using tweezers. Fifteen Petri dishes were prepared and labeled for the experimental groups (three dishes per group, with five larvae per dish). Larvae were injected into the last proleg using an insulin syringe. The larvae from the first two groups were injected with 10 µL of 0.9% NaCl solution and 5 µL of *P. aeruginosa* suspension, respectively. For larvae in the treatment group, the syringe was first loaded with 5 µL of *P. aeruginosa* suspension followed by 5 µL of phage suspension. After injection, larvae were placed in their designated Petri dishes. All dishes were transferred to a cardboard box with air holes and incubated in the dark at 37 °C. The protocol for *Z. morio* infection was identical to that for *G. mellonella*, except the volumes were doubled.

Larvae were monitored periodically for survival. *G. mellonella* were scored at 24 and 48 h post-infection, while *Z. morio* were observed daily for 5 days. Statistical analysis was performed using R ‘survival’ package.

### 2.8. Bioinformatic Analysis

Reads were trimmed using fastp 0.24.0 [43] and assembled with Unicycler 0.5.0 [44]. Phage termini were determined using PhageTerm 1.0.12 available on Galaxy Pasteur server [45].

Coding sequences were identified using Pharokka 1.7.5 [46]. Coding sequences coordinates were manually curated. Functional annotation of the predicted genes was performed using a combination of sequence similarity and structure-based approaches. HHpred searches were performed against the PDB70_mmCIF70_30_Mar, PfamA-v37, UniProt-SwissProt-viral70_3_Nov_2021, and NCBI_Conserved_Domains(CD)_v3.19 databases [47]. Foldseek and Dali searches [48,49] were performed using structures predicted by AlphaFold3 (https://alphafoldserver.com, accessed on 1 November 2025) [50], the structural alignments were manually curated. Default settings were used for all search engines. Local structure searches for pairwise protein comparisons as well as UPGMA tree construction based on structural data were performed using DaliLite.v5 [51]. Genome map was visualized with clinker [52].

Intergenomic similarities of phages were calculated using VIRIDIC web server (http://rhea.icbm.uni-oldenburg.de/VIRIDIC/, accessed on 1 November 2025) [53]. Protein and nucleotide alignments were made using Muscle5 [54]. Phylogenetic analysis of the aligned protein sequences was performed using IQ-TREE 2.4.0 [55] with ModelFinder [56] to determine the optimal substitution model.

In silico serogroup prediction of *P. aeruginosa* strains was performed using pasty tool [57], implementing locus database of Past 2.0 [58]. Defense systems were searched using DefenseFinder (v1.2.0) [59], only complete systems (non separate HMMer hits) were analyzed. Enrichment of defense system subtypes in O11 strains or a total set of Adele-resistant strains was assessed using Fisher’s exact test with Benjamini–Hochberg correction for multiple comparisons. The significance level was set at 0.05.

## 3. Results

### 3.1. Virion Structure and Growth

**Figure 1:**
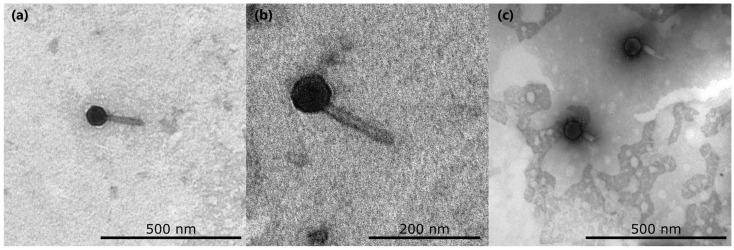
(**a**,**b**) Two representative electron microscopic images of phage Adele particles with non-contracted tails from two distinct biological replicates. (**c**) Electron microscopic images of phage Adele with contracted tails.

Transmission electron microscopy revealed that phage Adele displays the architectural features of myoviruses, with an icosahedral head of 76.0 ± 11.9 nm in apex diameter and a contractile tail of ~123.9 ± 21.7 nm in length (Figure 1).

**Figure 2:**
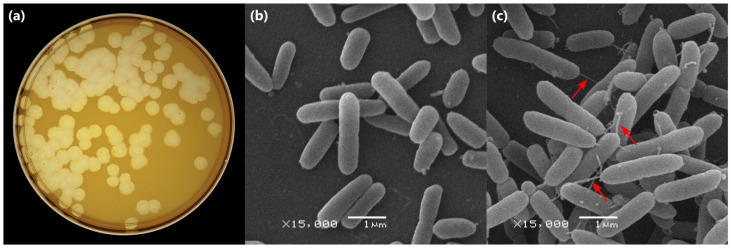
(**a**) Plaques formed by phage Adele on a double-layer agar plate. (**b**) Scanning electron microphotograph of a control sample of *P. aeruginosa* PAO1. (**c**) Scanning electron microphotograph of *P. aeruginosa* PAO1 incubated with phage Adele at MOI 100. Putative nanotubules are designated by arrows.

When plated using a double-layer agar assay with *P. aeruginosa* PAO1 as the host, phage Adele formed large clear plaques, a hallmark of potent lytic phages (Figure 2a). Infection of PAO1 with Adele induced prominent morphological changes, notably the formation of nanotubules with predominantly polar localization. These structures are known markers of local cell wall disintegration in dying cells (Figure 2b,c) [60].

### 3.2. Phage Reproduction Characterization

**Figure 3:**
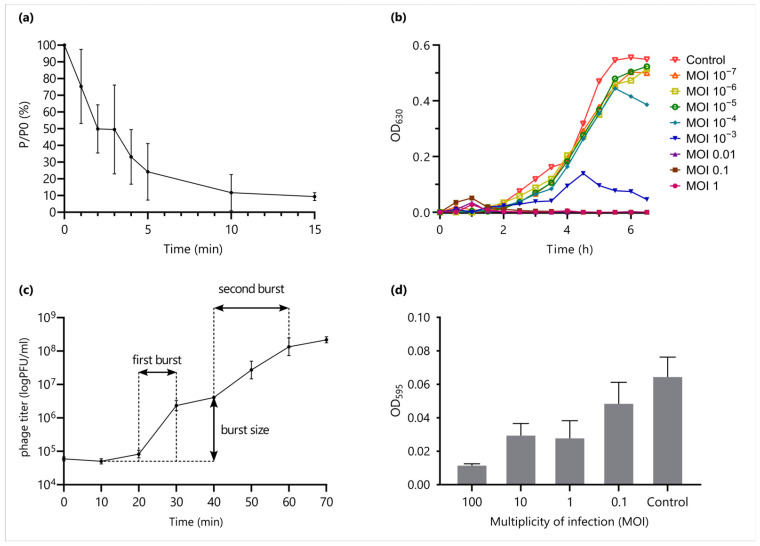
(**a**) Adsorption curve of phage Adele at the surface of *P. aeruginosa* PAO1 at MOI = 0.001. (**b**) Time-bacteriostatic dynamics curve. (**c**) One-step growth curve of phage Adele using *P. aeruginosa* PAO1 as the host strain at MOI = 0.01. (**d**) Concentration dependence of biofilm disruption by phage Adele. Error bars represent standard deviation.

The graph of adsorption rate showed rapid adsorption of phage Adele to its host bacterium, with approximately 75.6% of phage particles attached to the host after 5 min and 90.6% after 15 min (Figure 3a).

The growth curve of the *P. aeruginosa* control group in LB broth exhibited a typical sigmoidal shape (Figure 3b). The addition of phage Adele at different MOIs resulted in heterogeneous growth dynamics. In the high MOI groups (MOI 1–0.01), bacterial growth was completely suppressed during the initial stage (0–1.5 h), indicating immediate phage dominance. However, after 2 h, a gradual resurgence in bacterial growth was observed across these groups. In the middle MOI groups (MOI 10^−3^–10^−4^), a transient increase in bacterial concentration occurred initially (0–4.5 h), reflecting a temporary advantage for the host bacteria. This was followed by a distinct inflection point between 4.5 and 5.5 h, marked by a sharp decrease in bacterial load, indicating that the phage established a secondary infection advantage through propagating lytic cycles. In the low MOI groups (MOI 10^−5^–10^−7^), bacterial growth was similar to the control but exhibited a prolonged lag phase.

Obtained values were used to calculate local virulence values at different MOI (Table 1). The integrated value of virulence index, obtained for the MOI range from 10^−7^ to 1 and calculated from the onset of infection to the onset of the stationary phase in the phage-free control, was 0.535, similar to the value reported for phage T4 infecting *E. coli* [34]. Using the calculation for extended timeline and a shorter MOI range (from 10^−4^ to 1) previously used for *P. aeruginosa* phages [27], we obtained a high virulence index value 0.826. This establishes Adele as a highly virulent phage, with virulence comparable to other members of the *Pakpunavirus* genus [27].

According to the one-step growth curve, the latent period of phage Adele was 20 min following rapid rise period of 10 min and plateau was reached at 30 min after the start of experiment (Figure 3c). Then, the second burst of growth was observed; however, the growth rate was reduced and second rise period was 20 min. The second plateau was reached at 60 min. The calculated burst size was 59 ± 11 phage particles per infected cell after the first burst.

Phage treatment during biofilm formation significantly reduced biofilm biomass. The inhibition rate demonstrated a clear concentration-dependent effect; as the MOI increased from 0.1 to 100, the percentage of inhibition rose from 24% to 82%, indicating more efficient suppression at higher phage concentrations (Figure 3d).

### 3.3. Phage Stability

**Figure 4:**
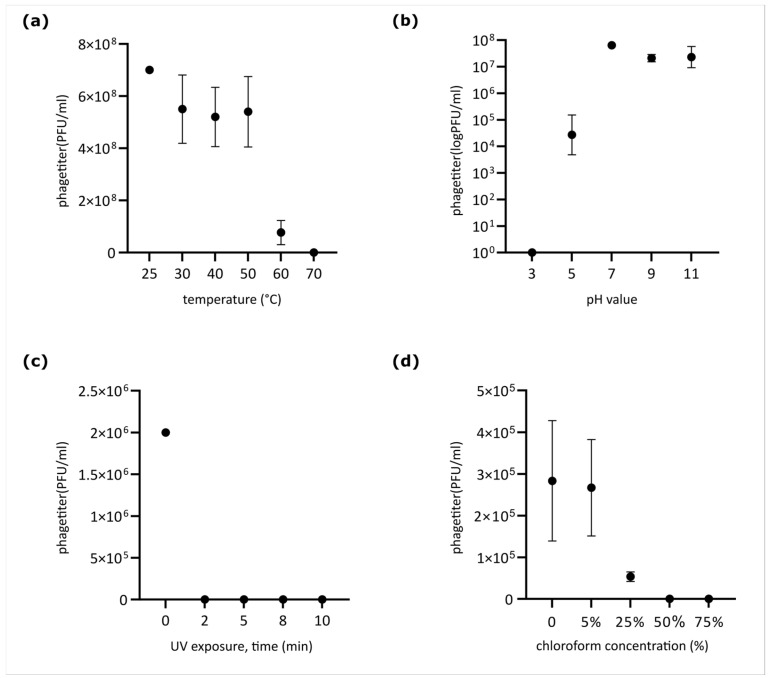
Stability of phage Adele under different conditions. (**a**) Temperature stability (25–70 °C); (**b**) pH stability (3–11); (**c**) UV resistance (0–30 min exposure); (**d**) Chloroform resistance analysis (0–75% *v*/*v*). Error bars represent standard deviation.

Phage Adele exhibited distinct stability profiles under various physicochemical stresses. Thermostability assays revealed no significant titer reduction after 1 h of incubation at temperatures ranging from 30 to 50 °C. However, the titer decreased gradually at higher temperatures, dropping by 2 log_10_ at 60 °C and leading to complete inactivation at 70 °C (Figure 4a). In pH tolerance tests (Figure 4b), the phage retained high infectivity within a pH range of 7.0 to 11.0. A significant titer reduction was observed at pH 5, and complete inactivation occurred at the highly acidic pH of 3. The phage was highly sensitive to UV radiation, losing all activity within 2 min of exposure (Figure 4c). Incubation with 25% chloroform reduced the initial phage titer by 1 log_10_. Complete inactivation was observed at chloroform concentrations of 50% and 75% (*v*/*v*) (Figure 4d)**.**

### 3.4. Testing Phage Activity in Vivo Using Galleria Mellonella and Zophobas Morio Models

**Figure 5:**
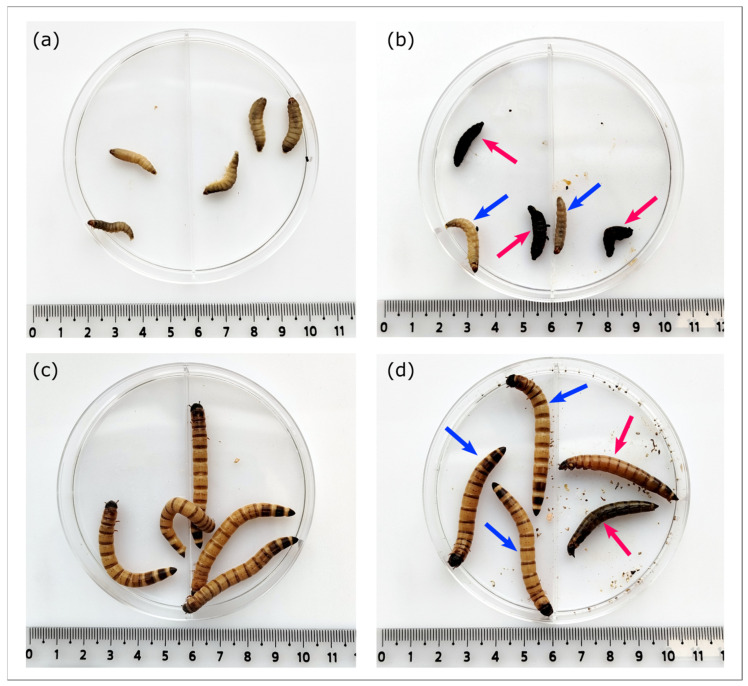
In vivo infection model in *Galleria mellonella* and *Zophobas morio* used for testing phage Adele. (**a**) *G. mellonella* larvae before injection (five live larvae); (**b**) *G. mellonella* larvae 24 h after injection with *P. aeruginosa* (10^6^ CFU/mL); (**c**) *Zophobas morio* larvae before injection (five live larvae); (**d**) *Z. morio* larvae 24 h after injection with *P. aeruginosa* (10^7^ CFU/mL). Blue arrows indicate live larvae, red arrows indicate dead larvae, which were identified by complete melanization and/or absence of movement. These examples illustrate how survival was assessed in the in vivo models described in Section 3.4.

In this study, the protective effects of phage Adele were evaluated using a therapeutic intervention model in *G. mellonella* and *Z. morio* larvae (Figure 5). *G. mellonella* is widely recognized as a relevant model for studying bacterial infections and phage therapy due to its mammalian-like innate immune system, ease of maintenance, and the ability to rapidly screen for survival [61]. *Z. morio* was chosen as an additional model due to its larger size, which allows for more precise injections and long-term observations, and to compare phage efficacy in an organism with slightly different physiology and immune response [62].

**Figure 6:**
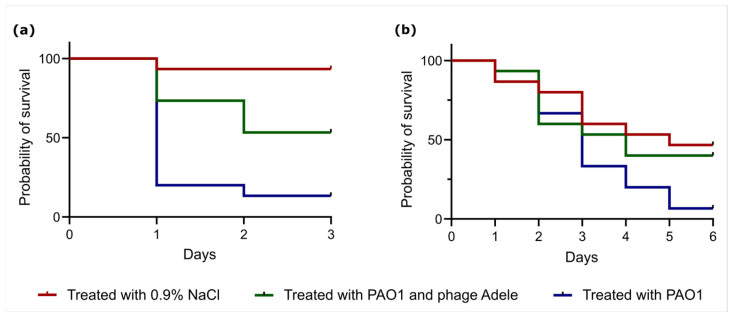
(**a**) Survival curves of *Galleria mellonella* infected with *P. aeruginosa* and treated with *Pseudomonas* phage Adele. (**b**) Survival curves of *Zophobas morio* infected with *P. aeruginosa* and treated with *Pseudomonas* phage Adele.

In the *G. mellonella* model, co-administration of phages with bacteria at an MOI of 1000 significantly improved larval survival (log-rank test, *p* = 0.0081). The protective effect was rapid, with a survival rate of 73% in the phage-treated group versus 20% in the bacteria-only group at 24 h. This difference persisted at 48 h, with survival rates of 53% and 13%, respectively (Figure 6a).

In contrast, the effect in the *Z. morio* model was less prominent, and the difference in survival curves did not reach statistical significance (log-rank test, *p* = 0.11) under the standard assumption of proportional hazards. At 48 h, survival was 60% in the phage group compared to 73% in the infected control. However, an apparent therapeutic effect emerged by day 5, with the phage-treated group exhibiting a 40% survival rate compared to the 6% survival in the bacteria-only group. This pattern hints at possible differences in the late effects (Fleming-Harrington G-rho family test, weighting parameter rho = −1, *p* = 0.03) but not in early effects (rho = 1, *p* = 0.3), requiring further validation (Figure 6b).

### 3.5. Phage Host Range

The host range of phage Adele was qualitatively characterized by testing its lytic activity against 39 *P. aeruginosa* isolates, including 26 clinical isolates with previously sequenced genomes and the *P. aeruginosa* PAO1 type strain (Supplementary Table S1). The phage lysed 15 of the 39 isolates. According to in silico serotyping of the sequenced genomes, Adele’s lytic activity varied by serogroup: it infected the single O1 isolate, one of three O3 isolates, three of five O5 isolates, and three of eight O6 isolates. In contrast, it failed to lyse the single O2 isolate and all eight O11 isolates.

No lytic activity was observed against three strains of *Pseudomonas savastanoi* and one strain of *Pseudomonas protegens.*

To elucidate the determinants of phage host range, we replicated a genomic search for phage defense systems across the tested strains with sequenced genomes previously described in [40]. The examined O11 isolates were enriched in defense system subtypes AbiE, Prometheus, RM_Type_IIG, RM_Type_IIG_2, and RloC compared to non-O11 strains (Fisher’s test, *p* < 0.05). However, none of these systems were universally present across all eight O11 genomes. Furthermore, no defense systems were overrepresented in a broader comparison of all Adele-insensitive strains to all sensitive ones (Fisher’s test, *p* < 0.05). Consequently, the shared resistance of these isolates to phage Adele is likely dictated by the O-specific antigen, which appears to be the principal determinant of infection, while the defense systems may instead have a more subtle effect on phage propagation efficiency.

### 3.6. General Genomics and Taxonomy

The *Pseudomonas* phage Adele genome comprises a double-stranded DNA molecule with direct terminal repeats of 1165 bp and 90,872 bp non-repeated sequence (NCBI Accession PV469300). The GC-content of the genome is 49.2%, much lower than ~67% in *P. aeruginosa.* This low GC-content is characteristic of phages in both *Skurskavirinae* and *Gorskivirinae* subfamilies of *Vandenendeviridae* family [63].

**Figure 7:**
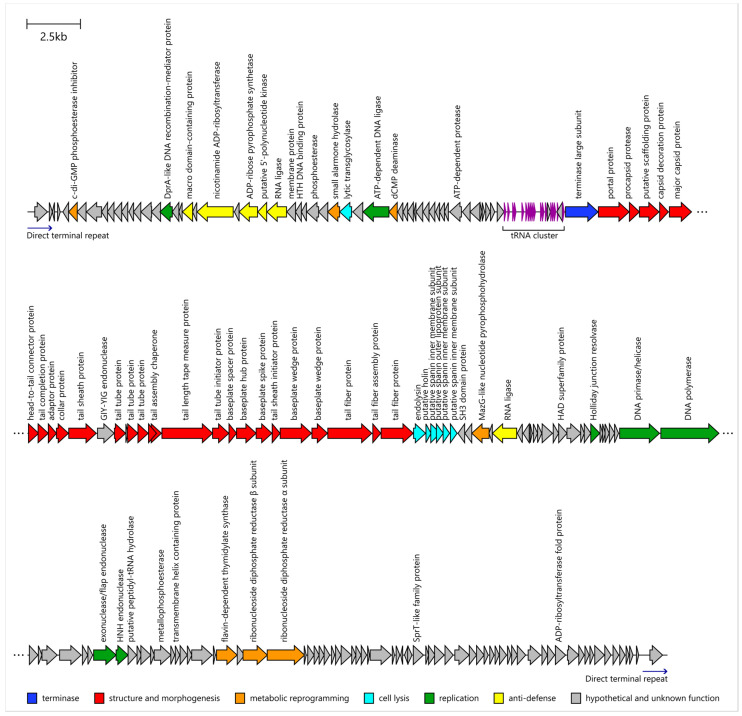
Genome map of *Pseudomonas* phage Adele. Coding sequences are colored based on their general functions, arrows directions represent the direction of transcription.

Consistent with typical phage genome organization, phage Adele sequence exhibits a modular architecture, with clearly defined structural gene module, lysis module and tRNA gene cluster (Figure 7). Genome does not contain known antibiotic resistance or virulence factor genes. The absence of encoded integrases or C repressors is consistent with an obligately lytic infection cycle.

**Figure 8:**
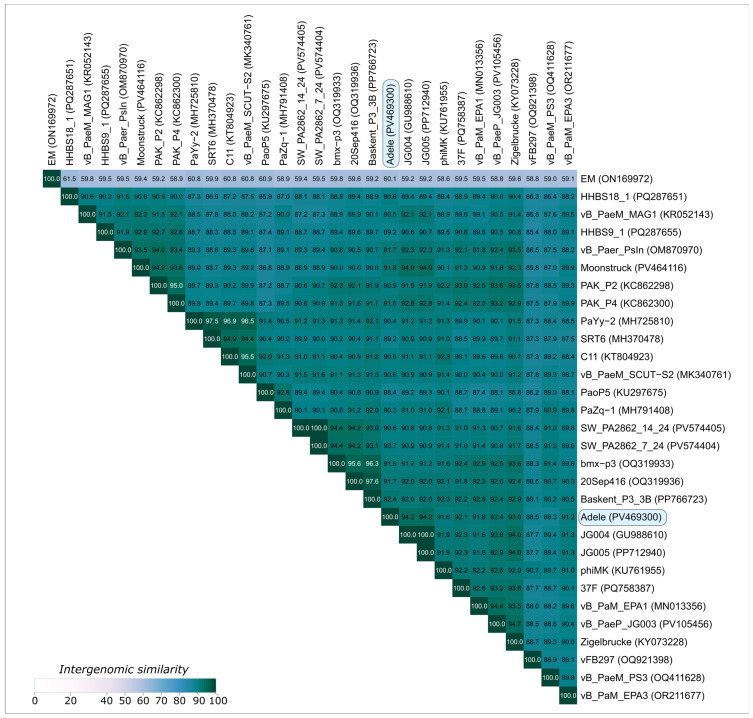
VIRIDIC heatmap generated using genomic sequences of the closest related *Pakpunavirus* phages and *Pseudomonas* phage EM (genus *Baldwinvirus)*. Phage Adele is highlighted.

VIRIDIC analysis of the top BLASTn 2.12.0+ search hits against the core_nt database demonstrated that the closest relatives of phage Adele are nearly identical *Pseudomonas* phages JG004 and JG005 (isolated in Braunschweig, Germany), belonging to the species *Pakpunavirus* JG004 (Figure 8). Phage Adele shares 94.2% intergenomic similarity with these phages, less than the 95% threshold proposed for species delineation. Therefore, we propose that *Pseudomonas* phage Adele represents a novel species within the genus *Pakpunavirus* according to current recommendations.

### 3.7. Phylogenetic Analysis of Conserved Proteins

Phylogenetic analysis of conserved proteins homologous to phage Adele was performed using amino acid sequences of major capsid proteins (MCPs), the ATPase and endonuclease domains of the large subunit of the terminase (TLS, terminase large subunit), and portal proteins (PPs). These homologs were identified by BLAST 2.12.0+ searches against the NCBI Genbank *Caudoviricetes* database. Additionally, a separate structural phylogeny was inferred using DALI, based on both predicted and experimentally determined structures of MCP and related HK97-like proteins.

**Figure 9:**
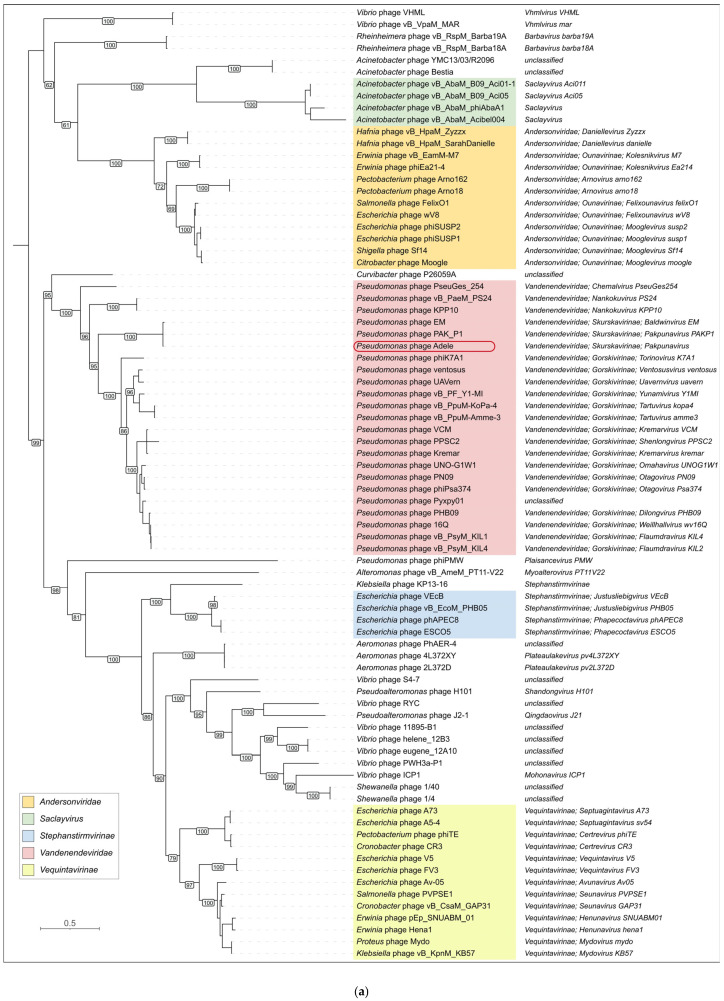

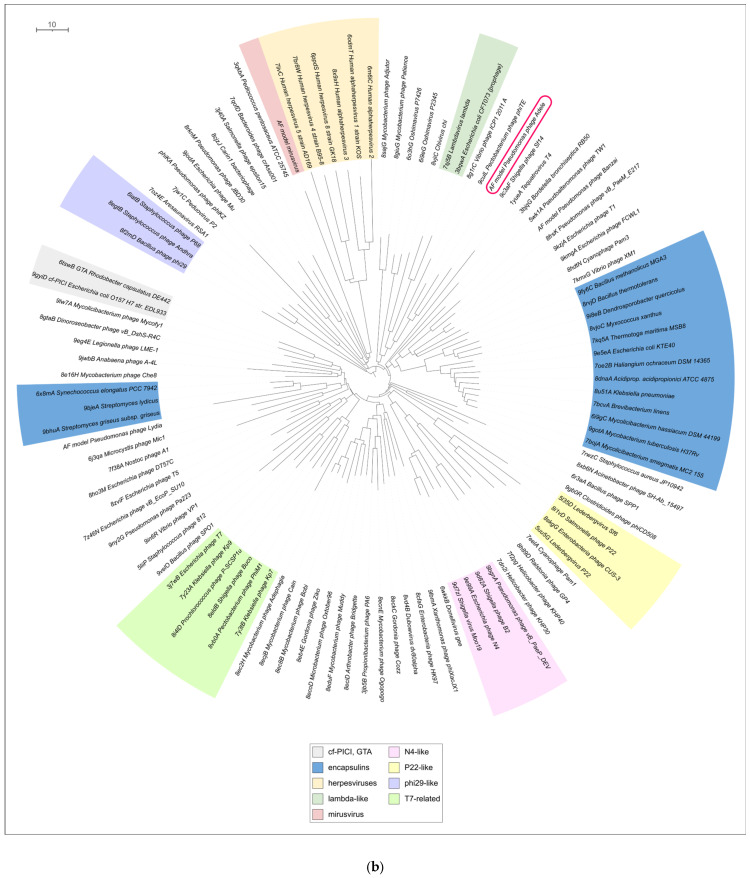
(**a**) Maximum-likelihood phylogenetic tree inferred from 80 representative amino acid sequences of the major capsid protein (MCP). Taxonomic assignments are shown in the tip labels and color-shaded blocks in the legend; binomial genus–species names are used for ICTV-classified phages, whereas NCBI annotations are used for unclassified phages. Phage Adele is highlighted with a red rounded box. Bootstrap support values are shown next to the corresponding nodes. The scale bar indicates the expected number of substitutions per site, and the tree is midpoint-rooted. (**b**) DALI-based similarity tree of HK97-fold proteins reconstructed from pairwise structural comparisons of major capsid proteins and related HK97-like proteins, including encapsulin shell proteins. Colored sectors correspond to the groups indicated in the legend. Phage Adele, represented by its AlphaFold3 MCP model, is highlighted with a red rounded box. PDB accessions are shown in the tip labels. The scale bar corresponds to the DALI Z-score, and the tree is midpoint-rooted.

The maximum-likelihood tree inferred from 80 representative MCP amino acid sequences recapitulates the currently recognized taxonomy of tailed phages (Figure 9a). Distinct, well-supported clades correspond to the family *Andersonviridae*, which comprises different *Enterobacterales*-infecting phages, and the family *Vandenendeviridae*, which comprises *Pseudomonas*-infecting phages, as well as to the subfamilies *Stephanstirmvirinae* and *Vequintavirinae* and to the genus *Saclayvirus*, which comprises *Acinetobacter*-infecting phages. Within *Vandenendeviridae*, MCP sequences further segregate into several lineages that match the currently accepted genera and subfamilies: members of *Skurskavirinae* (including *Pakpunaviruses*) form a compact group that is clearly separated from the more diverse *Gorskivirinae* clade. The *Skurskavirinae* clade contains tightly clustered phages of the *Baldwinvirus* and *Pakpunavirus* genera. Notably, *Andersonviridae* and *Saclayvirus* are themselves related and form a clade that is evolutionarily related to the *Vandenendeviridae* family. As expected, the tree based on similarity of the modeled structures of MCPs of phage Adele (as well as several previously published *Pseudomonas* phages) is consistent with the structural similarity of MCPs inferred from the sequence homology search: phages Adele (*Vandenendeviridae* family), Sf14 (*Andersonviridae* family) and φTE (*Vequintavirinae* subfamily) cluster together (Figure 9b). Beyond the similarities in MCPs, these three phages share commonalities in myovirus virion structure and seemingly lytic infection cycle [64,65]. Furthermore, all three phages possess a relatively developed replication apparatus, their capsids are cemented with decoration proteins, and their genomes are larger than average in size, although they differ notably from each other (the genome size of phage Sf14 is 88 kb, whereas that of φTE is 142 kb). Unfortunately, DALI structural comparisons lack statistical support, hindering reliable prediction of evolutionary relatedness, especially at deep nodes. Nevertheless, the DALI tree places another lytic myovirus, *Vibrio* phage ICP1_2011_A, at the beginning of the branch that also contains φTE, Sf14 and Adele. Phage ICP1_2011_A is known for donating its MCPs to a satellite PLE (phage-inducible chromosomal island-like element) [66]. Interestingly, this branch containing lytic myoviruses (Adele, Sf14, φTE, ICP1_2011_A) neighbors an earlier diverged group of evolutionarily related [67] enterobacterial siphoviruses (the temperate phage λ and the apparently lytic chi [68,69]). Also, at larger evolutionary distances from phage Adele, analysis of the DALI tree indicates a number of notable features:

- As expected, the DALI search found similar (Z-score > 4) proteins among HK97-fold capsid proteins and encapsulin shell proteins, except for an interesting case of a protein of unknown function from the Pfam DUF74 family of *Pediococcus pentosaceus* (Z-score 4.6), which was published as a pentameric structure (https://www.rcsb.org/structure/3QKB, accessed on 1 November 2025), in which the fold of each identical monomer is similar to that of the P-subdomain of HK97 capsids;
- Other phages infecting *Pseudomonas* are placed distantly from phage Adele;
- Encapsulin shell proteins cluster into two distant, distinct groups, supporting the polyphyletic and complex origin of encapsulins [70,71]: one group includes diverse Gram-positive and Gram-negative bacteria, whereas the other comprises *Streptomyces* and *Synechococcus*;
- A relatively recently discovered mirusvirus (the sequence from a nearly complete genome published in [72] was used for structural modeling) is clustered close to herpesviruses, branching somewhat earlier;
- Surprisingly, the capsid protein of a phage-like particle cf-PICI (capsid-forming phage-inducible chromosomal island) from *Escherichia coli* [73] forms a distinct branch together with the capsid protein of the gene transfer agent (GTA) from *Rhodobacter capsulatus*.

**Figure 10:**
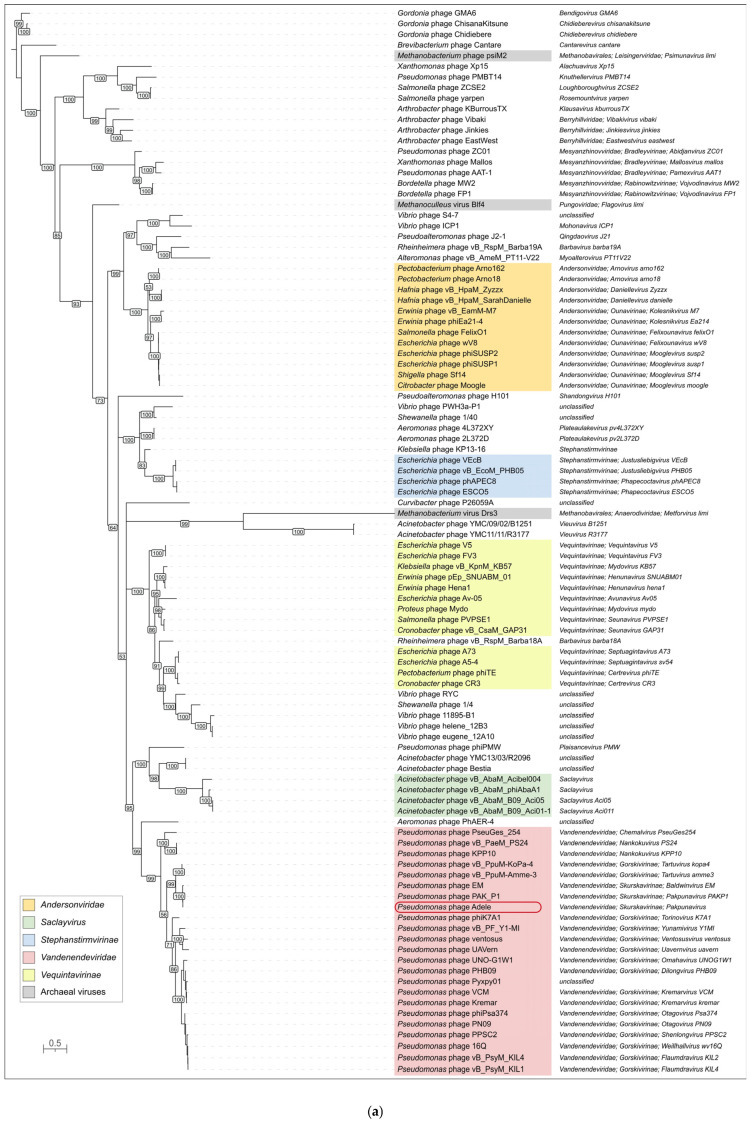

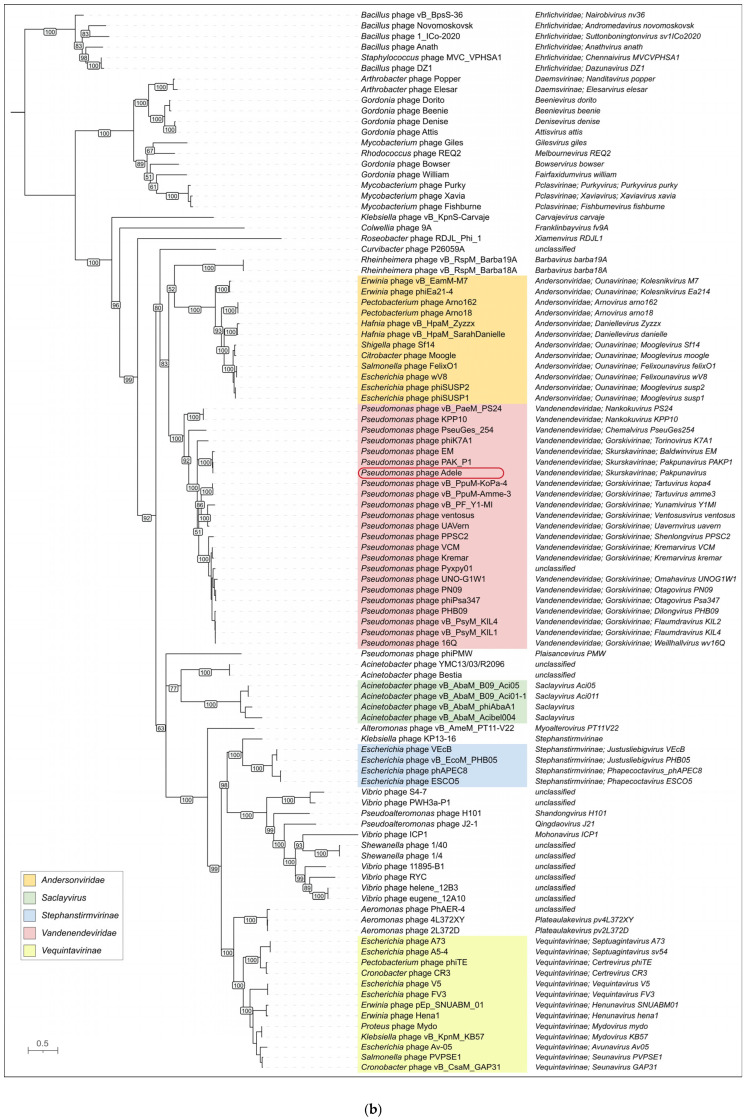
Maximum-likelihood phylogenetic trees inferred from 100 representative amino acid sequences of the ATPase domain of (**a**) the terminase large subunit, rooted to *Gordonia*-infecting phages (genera *Chidieberevirus* and *Bendigovirus*) and (**b**) portal protein, rooted to *Bacillales*-infecting phages (family *Ehrlichviridae*). Taxonomic assignments are shown in the tip labels and color-shaded blocks in the legend. Phage Adele is highlighted with a red rounded box. Bootstrap support values are shown next to the corresponding nodes; nodes with bootstrap support lower than 50% are collapsed into polytomies. The scale bar indicates the expected number of substitutions per site.

Phylogenetic analysis of the large subunit of the terminase was conducted using separate ATPase and endonuclease domains, located at the N-terminal and C-terminal ends of the protein, respectively, which were extracted from 100 sequences representing different taxa and unclassified phage groups. The topologies of the corresponding trees (Figure 10a and Supplementary Figure S1) are not strictly congruent, and the bootstrap support in the nuclease tree was generally lower. These patterns are consistent with subdomain-level horizontal transfer and with faster divergence of the nuclease domain relative to the ATPase domain. However, both trees cluster interrelated phages in a similar way and group representatives of the *Vandenendeviridae* family in a distinct clade, located not far from *Saclayvirus* and *Andersonviridae* phages. Adele’s branch is nested within the *Pakpunavirus* genus cluster, consistent with its classification as a novel species of that genus. The branch lengths within the *Pakpunavirus* ATPase TLS clade are short, reflecting the close evolutionary distance among these *Pseudomonas*-infecting myoviruses, and this phylogenetic placement mirrors the MCP tree described previously. Surprisingly, a BLAST search found distinct homologs not only among bacteriophages, but also among several archaeal tailed viruses of the *Methanobavirales* order and the *Pungoviridae* family, which is not assigned to an order. The ATPase and endonuclease trees place archaeal viruses differently: in the ATPase tree they fall into three distant branches, whereas in the nuclease tree they form three successive branches that still do not constitute a single monophyletic clade. Interestingly, a BLAST search using the Adele portal protein (PP) sequence did not find archaeal homologs with E-value < 0.05 in the NCBI Genbank *Caudoviricetes* database, although it still detected homologies with a number of the same groups (e.g., *Andersonviridae*, *Saclayvirus*, *Stephanstirmvirinae*, *Vequintavirinae*) as in the cases of MCP and TLS. Importantly, the PP tree (Figure 10b) has a topology different from the MCP and TLS trees, while still placing the *Vandenendeviridae* representatives in a distinct clade and phage Adele within this clade, in a subclade together with *Pseudomonas* phage PAK_P1 (*Pakpunavirus PAKP1*).

### 3.8. Analysis of Structural Genomic Module

#### 3.8.1. Comparative Analysis with Related Taxa

The genomic region encoding the structural proteins is syntenic within the *Pakpunavirus* genus as well as the closest related genus *Baldwinvirus* (Supplementary Figure S2). Notably, phage Adele contains a putative GIY-YIG homing endonuclease gene (PaAdele_069) inserted between the genes encoding the tail sheath and tail tube proteins. This insertion is shared between several *Pakpunavirus* genomes (e.g., JG004, JG005, PAK_P4, PaZq-1) but appears to be a rare derived trait within the genus.

Up to date, there are no detailed structural studies of *Pakpunavirus* virions. However, low-resolution Cryo-EM images are available for ΦPsa374 (species *Otagovirus* Psa374), a phage of the same family *Vandenendeviridae* [74]. Furthermore, the high-resolution Cryo-EM studies were performed on *Shigella* phage Sf14 (*Mooglevirus Sf14*), belonging to the related family *Andersonviridae* [64], and on *Pectobacterium* phage φTE (*Certrevirus φTE*) of the subfamily *Vequintavirinae* [65].

**Figure 11:**
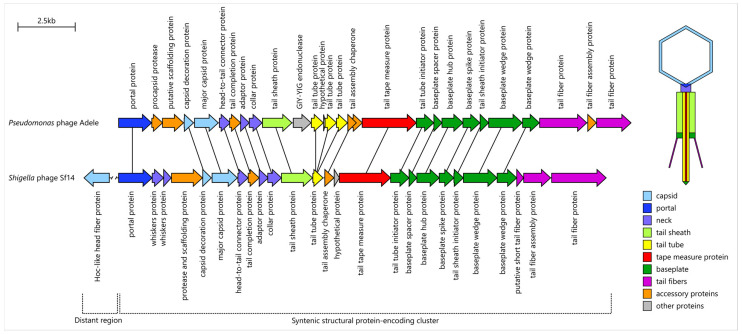
Structural gene clusters of *Pseudomonas* phage Adele (family *Vandenendeviridae*) and *Shigella* phage Sf14 (family *Andersonviridae*). Linkers connect best hits obtained using DaliLite.v5 pairwise alignments of AlphaFold3-preditcted structures with Z-scores > 7. Colors represent the functional roles of proteins within the phage particles.

A limited synteny is shared between Adele and aforementioned distant relatives (example in Figure 11), with nearly all structural proteins being orthologous, although their relatedness is masked by low sequence similarity. The primary differences lie in their accessory appendages, which are supposedly the late acquisitions. Specifically, compared to Sf14 phage Adele lacks a head fiber Hoc-like protein, putative short tail fibers and neck whiskers; unlike φTE it lacks decoration Pagoda protein and short tail fibers.

Based on genome annotation, the icosahedral capsid of phage Adele is formed from HK97-fold major capsid protein (PaAdele_063) stabilized by beta-tulip decoration protein (PaAdele_062) structurally related to Sf14 gp33, φTE φTE_212, YSD1 gp16 and P21 SHP protein. The capsid triangulation number remains unknown. However, we note that Adele head is closer in size (~76 nm, containing 93 kbp genome) to the T = 9 capsids of Sf14 (82 nm, 88 kbp) and Psa374 (80 nm, 98 kbp) than to the T = 13 capsid of φTE (105 nm, 142 kbp).

Baseplate-encoding region (PaAdele_078-PaAdele_084) consists of seven genes and is fully syntenic with the loci in Psa374 (gp74-gp80), Sf14 (gp44-gp50) and φTE (φTE_229-φTE_235), demonstrating one-to-one protein structural similarity despite significant sequence divergence. We propose that these phages share identical baseplate organization, described for φTE [65].

Tail sheath protein shares common fold in Adele, φTE and Sf14. It possesses one additional lateral domain, thus representing type 2 of contractile sheath proteins [75]. This domain consists of seven-stranded β-sandwich, flanked with an α-helix, does not participate directly in contraction [65] and is found not only in sheath proteins (PDB ID 9khy), but also in podovirus nozzle proteins (8i4m, 7ey9), auxiliary capsid proteins (7qof, 8giu), flagellar cap FliD proteins (6iwy, 5h5w) or flagellin (8erm).

#### 3.8.2. Tail Tube Proteins

**Figure 12:**
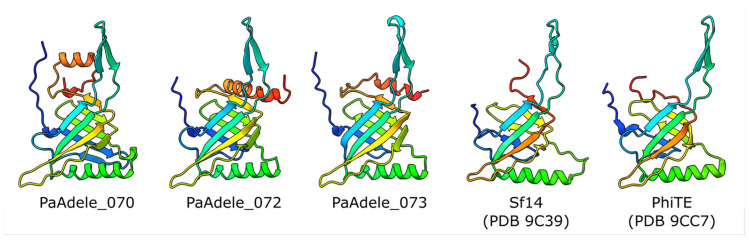
Structures of tail tube proteins encoded in phage Adele and related phages. Rainbow colouring uses a colour gradient where the N-terminal end is blue and the C-terminus is red.

All analyzed *Vandenendeviridae* genomes contained a cluster of three tail tube protein genes, which are putatively functionally specialized and positioned differently within the tail. In phage Adele these proteins share 24–29% identity, however their predicted AlphaFold3 structures were nearly identical (RMSD 2.18–2.3 in 141–143 aligned residues of total 159–174 residues) except for a variable C-terminal α- helix, which is not the part of the canonical major tube protein fold, and a loop after a first beta-strand (Figure 12).

**Figure 13:**
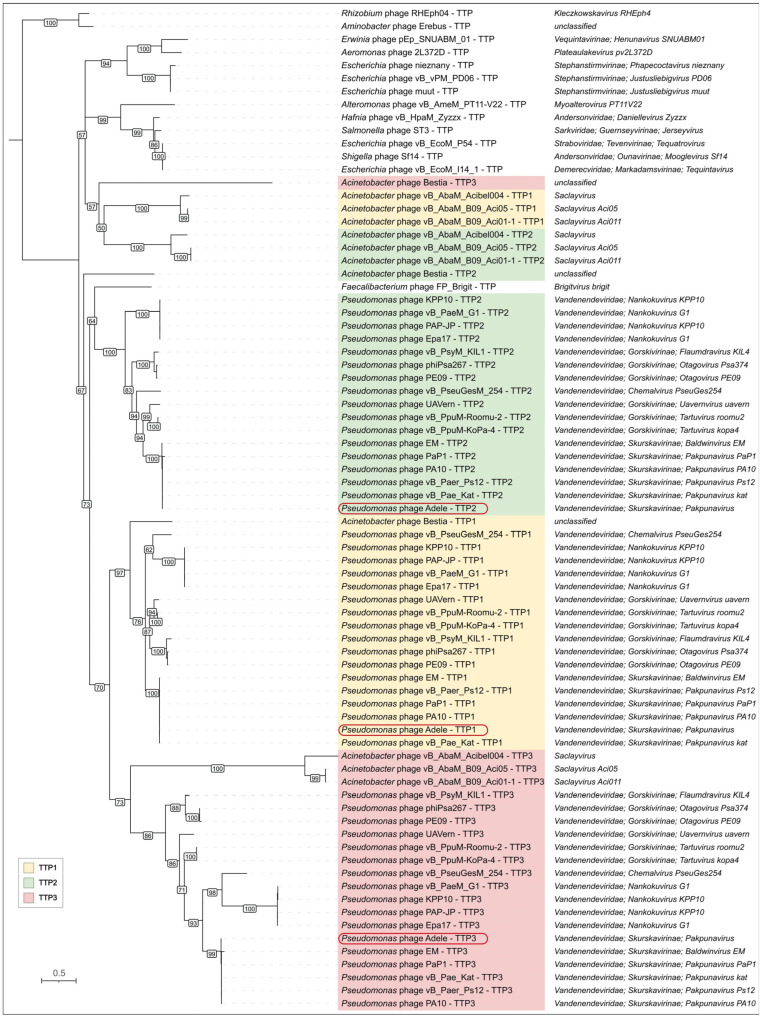
Maximum-likelihood phylogenetic tree inferred from 75 representative amino acid sequences of the tail tube protein. Taxonomic assignments are shown in the tip labels and color-shaded blocks in the legend. Phage Adele is highlighted with a red rounded box. Bootstrap support values are shown next to the corresponding nodes; nodes with bootstrap support lower than 50% are collapsed into polytomies. The scale bar indicates the expected number of substitutions per site, and the tree is rooted to *Rhizobium* phage RHEph04 and *Aminobacter* phage Erebus.

The adjacent positioning of these genes suggests that they originated from a single gene that underwent tandem duplications in an ancestral phage and subsequently diverged. This hypothesis is supported by phylogenetic reconstruction (Figure 13)—in members of the *Vandenendeviridae*, these three proteins, designated TPP1, TPP2, and TPP3, form distinct clusters on the maximum-likelihood evolutionary tree, indicating vertical transmission from the family common ancestor. However, the earlier stages of this locus’s evolution reveal a more intricate picture, pointing to the transfers of its fragments between progenitor lineages of modern-day *Pseudomonas* and *Acinetobacter* phages before the *Vandenendeviridae* family diverged.

#### 3.8.3. Tail Assembly Chaperone Locus in *Vandenendeviridae* and Related Phage Taxa

**Figure 14:**
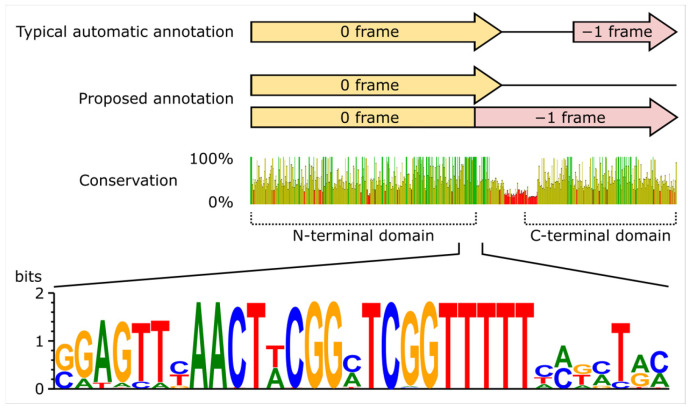
Tail assembly chaperone locus of *Pseudomonas* phage Adele. Arrows show the direction of transcription. Typical annotation in publicly available *Pakpunavirus* genomes is compared with proposed annotation containing −1 programmed ribosomal frameshift. Conservation of nucleotide positions is calculated using 56 unique allele sequences of this locus from 159 *Vandenendeviridae* phage genomes. Domain boundaries are determined based on AlphaFold3 Structure prediction. The conserved region containing suggested frameshift site is visualized using Weblogo3.

Tail assembly chaperone is encoded by the gene PaAdele_074 in the phage Adele. Immediately downstream, and upstream of the tape measure protein gene, lies a region that is routinely annotated in publicly available *Pakpunavirus* genomes as containing a gene encoding hypothetical protein. However, tail assembly chaperones function as a co-polymer of a major short (TAC-N) and a minor long (TAC-NC) isoforms. These isoforms are often produced via programmed ribosomal frameshift near the 3′-end of the TAC-N reading frame, which bypasses a stop codon and allows to translate TAC-NC protein [76]. We argue that this is the case in phage Adele, and TAC-N-encoding PaAdele_074 contains a −1 programmed ribosomal frameshift site, while the downstream open reading frame represents not a separate gene but a fragment of a C-terminal domain of the TAC-NC (Figure 14).

To test this hypothesis, we extracted 56 unique allele sequences of this locus from 159 *Vandenendeviridae* phage genomes. The resulting multiple sequence alignment revealed significant nucleotide divergence (42.47% minimal identity; 13.9% invariant sites). However, in every instance, the introduction of a −1 frameshift at the end of the gene encoding the TAC-N extended the open reading frame through the entire locus for over 200 bp. Importantly, not a single genome contained a premature stop codon in the −1 frame even in regions currently annotated as “intergenic”. We interpret this comprehensive genomic conservation as strong evidence for a programmed ribosomal frameshift at this locus.

Furthermore, the terminal region of the TAC-N gene exhibited notably higher sequence conservation in our alignment and contained an invariant GTTTTT motif. Similar polyT-containing sequences are known −1 frameshift signals (slippery sequences) in major coat protein gene of T7 phage and TAC-N genes of T4-like phages [76]. To assess the broader conservation of this signal, we inferred a multiple sequence alignment of the tail assembly chaperone region of 231 *Andersonviridae* family phages. This analysis revealed the same conserved GTTTTT motif positioned near the 3′ end of their respective TAC-N genes, already suspected as frameshift site in *Mooglevirus* genome annotations [77].

#### 3.8.4. Tail Fiber Proteins

BLAST 2.12.0+ searches identified homologs of tail fiber protein 1 (TFP1, PaAdele_085) with e-value < 0.05 among TFP1 proteins of all the genera within the *Vandenendeviridae* family. In more evolutionarily distant taxa beyond the genus *Pakpunavirus*, this homology tended to be limited to the N-terminal particle-binding domain, highlighting faster evolution of the receptor-recognizing parts of receptor-binding proteins and possible replacements of these modules.

No representatives of the evolutionarily related *Andersonviridae* or *Vequintavirinae* clades were found by this search. In contrast, distinct homologs of the Adele TFP1 N-terminal domain were identified among *Acinetobacter*-infecting phages of the *Saclayvirus* genus. The search also revealed more distant homology in the N-terminal parts of structural and supposed receptor-binding proteins of *Kyanoviridae* cyanophages, enterobacteria-infecting phages of the genus *Punavirus* (sensu lato “P1-like”, assigned directly to the class *Caudoviricetes*), *Straboviridae* phages (T4-like), and phages assigned to other taxa. Notably, this search did not detect similarity between TFP1 of phage Adele and TFP2 of Adele or other *Vandenendeviridae* phages, except in the case of *Pseudomonas* phage KPP10 (*Nankokuvirus KPP10*). In this case, similarity was found between a short KPP10 gene located immediately upstream of the KPP10 TFP2 gene and the N-terminal particle-binding domain of Adele TFP1. At the same time, KPP10 TFP2 is shorter than TFP2 proteins of other *Nankokuvirus* phages and apparently lacks the particle-binding domain. This is consistent with the assumption that the homologous upstream-located gene was acquired by KPP10 horizontally and functionally replaces the lost ancestral N-terminal domain.

**Figure 15:**
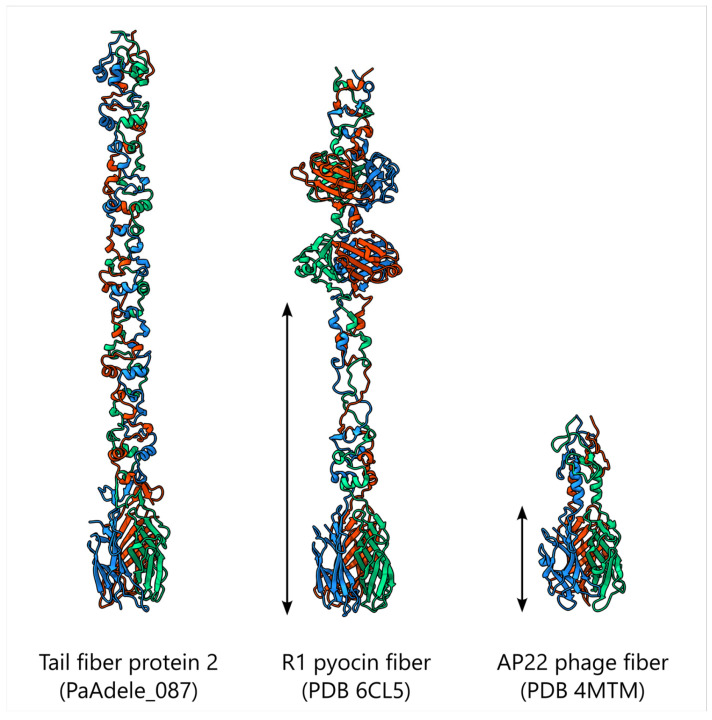
AlphaFold3 predicted homotrimeric structure of tail fiber protein 2 of phage Adele and related proteins from PDB database. Similar regions according to Foldseek search are marked with arrows. Each monomer is shown in different color.

For tail fiber protein 2 (TFP2, PaAdele_087) the most similar homologs were again found within the *Vandenendeviridae* family, but additional related groups included *Acinetobacter*-infecting phages of the *Saclayvirus* genus and phages infecting both Gram-negative and Gram-positive bacteria, including members of the *Chaseviridae* family. Unlike TFP1, a few representatives of the *Vequintavirinae* subfamily and the *Andersonviridae* family (a small fraction of all phages of these taxa contained in the NCBI Genbank *Caudoviricetes* database) were recovered by this search. As in the case of TFP1, sequence similarity in phages that are evolutionarily distant from Adele predominantly corresponded to the more conserved N-terminal particle-binding domain. Dali and Foldseek search in PDB database revealed that structural homologs of C-terminal particle-binding domain shared between Adele, pyocins of *P. aerguinosa* and tail fiber of *Acinetobacter baumannii* bacteriophage AP22, belonging to *Obolenskvirus* genus (Figure 15). This domain bind cations and putatively participates in binding to the carbohydrate portion of the lipopolysaccharide molecule [78].

### 3.9. Phage Metabolic Reprogramming and Anti-Defense Systems

Phage Adele possesses a set of accessory metabolic genes collectively facilitating thymidine production. They encode ribonucleoside-diphosphate reductase subunits (PaAdele_139–140; converts NDP to dNDP), dCMP deaminase (PaAdele_036; dCMP to dUMP) and flavin-dependent thymidylate synthase (PaAdele_137; dUMP to dTMP). The gene PaAdele_135 encodes a protein containing a C-terminal domain of unknown function DUF3310. The same domain is found in phage T7 kinase 1.7, catalyzing phosphorylation of dGMP and dTMP to the corresponding nucleoside diphosphates [79], hinting that the protein encoded by PaAdele_135 may have a nucleotide kinase function.

A gene PaAdele_032 encodes a small alarmone hydrolase, possessing conserved histidine-aspartate (HD)-domain fold. This family of proteins hydrolyzes hyperphosphorylated nucleotide messengers (p)ppGpp, a key global regulator of transcription in bacterial cells, and/or (p)ppApp, produced as byproduct by cell-killing T6SS effectors [80,81]. Reliable prediction of whether the Adele-encoded enzyme prefers (p)ppGpp or (p)ppApp is hindered by its low sequence similarity to characterized hydrolases [82]. We hypothesize that this protein may complement the chromosomally encoded promiscuous small alarmone hydrolase of *P. aeruginosa* during phage infection [83]. Another one gene (PaAdele_096) encodes a MazG-like nucleotide pyrophosphohydrolase, another candidate enzyme to cleave hyperphosphorylated nucleotide messengers [84]. However, an exact function can possibly vary within the family, as cyanophage MazG preferentially hydrolyze dGTP and dCTP but not (p)ppGpp [85], and we cannot exclude viperin-produced nucleotide derivatives as the primary targets [86].

Another signaling system in *P. aeruginosa* involves a cyclic dimeric guanosine monophosphate (c-di-GMP) which regulates motility and biofilm formation in response to environmental factors. The phage Adele encodes Dap1 (PaAdele_006), a characterized inhibitor of bacterial c-di-GMP phosphoesterase DipA [87]. Expression of Dap1 was shown to reduce bacterial swimming and swarming motility; it also protects the HNH endonuclease encoded by PaoP5 Orf50 from proteolytic degradation [87]. However, the latter enzyme, despite being described as important for DNA packaging in PaoP5, is not conserved within the *Pakpunavirus* genus and is absent in phage Adele.

A critical step in establishing infection involves overcoming cellular defense mechanisms. Phage Adele encodes two recently described enzymes that counteract NAD^+^ depletion triggered by abortive infection systems: ADP-ribose pyrophosphate synthetase (PaAdele_024) and nicotinamide ADP-ribosyltransferase (PaAdele_022). Together they form a NAD^+^ reconstitution pathway 1 capable of regenerating NAD^+^ from its hydrolysis products, nicotinamide and ADP-ribose [88].

A gene located downstream (PaAdele_020) contains a macro domain, known to bind ADP-ribose residues. It shares several key catalytic residues with the yeast protein Poa1p (His23, Gly33, Asn26; Ser30 is substituted with Thr), suggesting that this protein may perform hydrolysis in 1″-position of ADP-ribose derivatives [89], releasing free ADP-ribose for NAD^+^ reconstitution pathway 1. However, the corresponding natural substrates of Poa1p, ADP-ribose-1″-phosphate and 1″-O-Acetyl-ADP-ribose have not been previously found in bacteria. Nevertheless, we speculate that PaAdele_020 could potentially counteract anti-phage defense systems catalyzing ADP-ribosylation as an effector mechanism or producing ADP-ribose derivatives as secondary messengers.

Several bacterial phage defense systems, including PrrC, retron-Eco7 and PARIS, protect against viral infection by cleaving cellular tRNA molecules, thereby blocking translation. Phages can evade these defense systems by expressing their own tRNAs from strong promoters [90]; these phage-encoded tRNAs may also possess altered sequences conferring nuclease resistance [91,92]. Phage Adele encodes for a total of 17 putative tRNA genes in a compact genome cluster. Additionally, cleaved tRNA can be restored by the combined action of putative polynucleotide 5′-kinase (PaAdele_025), phosphorylating broken ends, and two distinct RNA ligases (PaAdele_026 and PaAdele_098), sealing nicks. Notably, unlike T4 phage, the first of the listed proteins lacks 3′-phosphatase domain.

Importantly, BLAST 2.12.0+ searches using the sequences of the aforementioned proteins revealed several distinct patterns:

- Protein PaAdele_006—homologs were found only in phages of the family *Vandenendeviridae*.
- Protein PaAdele_020—homologous proteins from *Salmonella* phages of the family *Demerecviridae* are more similar to the Adele protein than a substantial part of homologs from *Vandenendeviridae*. In addition, multiple *Straboviridae* phages also precede proteins from the evolutionarily related taxa identified using conserved protein sequences and discussed in Section 3.7.
- Protein PaAdele_022—homologous proteins from phages of the families *Herelleviridae*, *Ackermannviridae* and several other taxa are more similar to the Adele protein than those from the evolutionarily related taxa discussed in Section 3.7.
- Proteins PaAdele_024, 025, 026, 032, 096, 098, 135, 137, 139 and 140—BLAST searches generally produced patterns similar to those observed for 020 and 022, or yielded significant hits to phage proteins belonging to taxa that were not detected at all when searches were conducted using MCP and TLS sequences.

Taken together, these BLAST results indicate intense horizontal gene transfer and suggest that genes important for successful replication can persist in divergent lineages, contributing to the highly mosaic architecture of the Adele genome.

## 4. Discussion

### 4.1. Biological Properties, Host Range and Therapeutic Potential of Phage Adele

Adele combines fast adsorption with efficient replication and high suppressive activity against planktonic cultures, a kinetic profile typical of highly virulent caudoviricetes. Roughly 75.6% of particles adsorbed within 5 min and 90.6% within 15 min, indicating rapid recognition of the *Pseudomonas aeruginosa* surface and a short decision window before infection becomes irreversible. The isolated *P. aeruginosa* phage Adele showed short latent period and two bursts of progeny release, reaching a titer from 6 × 10^4^ up to 1.5 × 10^8^ PFU/mL within 60 min since the start of the experiment. The latent period of 20 min and the amount of progeny phages released after the first burst was 59 ± 11 which is similar to related *Pakpunavirus* phages PaP1 and HJ01 [15,93]. Such characteristics as short latent period and large burst size can be advantageous traits of phages belonging to *Pakpunavirus* genus in therapy. For example, the mentioned HJ01 phage showed its effectiveness in treating canine pyoderma caused by *P. aeruginosa*, showing faster skin surface recovery compared to non-treated control group [93].

In dynamic killing assays, high multiplicities of infection (MOIs) produced complete suppression of bacterial growth during the early phase, whereas intermediate MOIs led to delayed but pronounced inflection points in the growth curves, consistent with secondary infection cycles gradually dominating the population. When summarized by the virulence index, Adele reached 0.535 across an MOI range of 10^−7^–1 and 0.826 in the narrower range of 10^−4^–1, values comparable to those reported for classical model phages such as T4 and for highly lytic *Pseudomonas* phages in similar assays [27,34]. Together with a short latent period and large burst size, these data suggest that Adele can efficiently control bacterial populations across a broad spectrum of initial phage-to-bacterium ratios, which is important in therapeutic settings where the local MOI is difficult to predict.

In vitro, Adele also showed a pronounced antibiofilm effect. When present during biofilm formation, it reduced biomass in a concentration-dependent manner from 24% inhibition at MOI 0.1 to 82% at MOI 1000. These values fall within the range described for other lytic *P. aeruginosa* phages and confirm that complete eradication is rarely achieved by a single phage, even under favorable conditions [94]. Given that *P. aeruginosa* biofilms substantially reduce antibiotic penetration and shield bacteria from immune effectors, even partial reduction in biomass may be clinically meaningful, especially in combination with antibiotics, mucolytic compounds or additional phages targeting alternative receptors. The observation that substantial inhibition is already achieved at intermediate MOIs indicates that Adele could be a useful component of rationally designed cocktails aimed at different stages of biofilm development or distinct subpopulations in structured communities. The molecular contribution of Adele’s putative biofilm-modulating enzymes to this phenotype remains a prediction based on homology rather than a demonstrated mechanism in our system, and therefore cannot be formally confirmed at present.

Experiments in *Galleria mellonella* and *Zophobas morio* larvae provided a first indication of the therapeutic potential of Adele. In *G. mellonella*, co-injection of phage and bacteria at an MOI of 100 led to a rapid and sustained survival benefit: 73% of larvae in the phage-treated group were alive at 24 h compared to 20% in the infection control, and 53% versus 13% at 48 h (log-rank *p* = 0.0081). These data are in line with previous studies where successful phage therapy in this model depended on both rapid bacterial access and functional innate immune responses [95]. They indicate that, under permissive conditions, Adele can substantially reduce mortality within a clinically relevant time frame.

Adele lysed 15 of 39 *P. aeruginosa* isolates in our panel. The distribution of susceptible strains across O-serogroups points to the O-specific antigen (OSA) of the lipopolysaccharide as the primary receptor: Adele infected isolates of serogroups O1, O3, O5 and O6 strains, but failed to infect any of the O11 isolates. This pattern mirrors earlier studies where OSA structure proved to be the key determinant of susceptibility to *Pakpunavirus* phages [21,22,23,24]. Our data therefore support a model in which Adele’s tail fiber repertoire is tuned to a subset of OSA chemotypes, with serogroup O11 presenting an incompatible O-antigen that prevents adsorption.

Another potential explanation for the resistance observed in this serogroup could involve phage defense systems. The O11 isolates were enriched in several defense system subtypes, consistent with the tendency of defense islands to associate with particular clonal backgrounds [96]. However, none of these systems was universally present in all O11 isolates, and no single defense module was significantly overrepresented when all resistant strains were compared to all sensitive ones. This finding can be explained by the extensive arsenal of anti-defense systems encoded by phage Adele, which are capable of countering abortive infection scenarios such as cell death via NAD^+^ hydrolysis or tRNA cleavage. In addition, more anti-defense systems are likely hidden among hypothetical proteins. For example, we did not identify genes for adenine or cytosine methylation, a modification experimentally discovered in the related PaP1 phage [97] that may protect the genome against endonuclease-based defense mechanisms. Thus, the data argue against a simple scenario in which a dominant intracellular defense explains the observed resistance pattern. Instead, the findings favor a model where adsorption specificity, dictated by the O-antigen, represents the primary barrier, whereas intracellular defenses modulate burst size or replication success in a strain-specific manner.

From a therapeutic perspective, Adele’s host range reinforces the need to deploy it as part of cocktails that also cover O11 and other prevalent serotypes in clinical collections. At the same time, the modular architecture of Adele’s tail fibers—with conserved N-terminal particle-anchoring domains and variable C-terminal receptor-binding regions—suggests a potential route for host-range engineering by domain swapping or recombination, analogous to strategies already demonstrated for phages infecting *Enterobacterales* [98].

### 4.2. Genomic Architecture and Evolutionary Context of Phage Adele

Genomic analysis firmly places Adele within the family *Vandenendeviridae* while clearly differentiating it from previously described species. Its 92 kbp dsDNA genome with 1165 bp direct terminal repeats, 49.2% GC-content and a modular organization comprising structural, lysis and tRNA gene clusters fits well within the spectrum of *Pakpunaviruses* and related *Vandenendeviridae* phages. VIRIDIC analysis revealed 94.2% intergenomic similarity to *Pseudomonas* phages JG004 and JG005, below the 95% species demarcation threshold, supporting the designation of Adele as a novel species within the genus *Pakpunavirus* [99]. Importantly, the genome lacks identifiable integrase genes, canonical toxin genes and known antibiotic resistance determinants, which is consistent with an obligately lytic infection cycle and favorable from a safety perspective.

The structural module of Adele is strongly conserved and syntenic with closely related *Pakpunaviruses*, more distant members of *Vandenendeviridae*, and myoviruses such as *Shigella* phage Sf14 and *Pectobacterium* phage φTE. Structural comparisons of the major capsid protein place Adele within the canonical HK97-fold capsid protein radiation, which encompasses numerous tailed phages and underlies a wide variety of particle sizes and morphologies [100,101]. The conservation of head and tail gene order across *Vandenendeviridae* suggests that the core morphogenesis apparatus has been shaped predominantly by vertical inheritance, with only occasional recombination events.

Two features of the tail region are particularly informative. First, the triplicated tail tube locus forms three distinct but closely related clades within *Vandenendeviridae*, implying that local duplication and putative functional divergence of a tail tube ancestor occurred before the divergence of *Vandenendeviridae* and that copy number has been stably maintained since. Second, the tail assembly chaperone locus exhibits a conserved −1 programmed ribosomal frameshift, with a shared GTTTTT slippery sequence at the 3′ end of the TAC-N gene and uninterrupted reading frames in the −1 phase across divergent genera. The discovery of a similar motif in related *Andersonviridae* clade suggests that this translational strategy has been retained over long evolutionary timescales. These “frozen accidents” in gene organization and decoding contrast with the higher volatility of peripheral components such as tail fibers and head appendages and highlight how certain architectural solutions can persist even in highly modular and rapidly evolving phage genomes.

Different groups of *P. aeruginosa* phages employ distinct strategies to take over host metabolism, ranging from simple resource depletion to active pathway modulation [102]. Specifically, metabolomic changes during *Pakpunavirus* infection have been previously studied in a system “PA1 strain—PaP1 phage” [103]. Although delineating phage-caused metabolic reprogramming from host anti-phage defense mechanisms is challenging, the most prominent effect of the PaP1 enzymes is a significant increase in thymidine concentration. Adele shares with PaP1 a thymidine-production module to expand DNA precursor pools for replication. Putative nucleotide kinase (PaAdele_135) may also participate in securing precursor supply under fluctuating host conditions. Free ribonucleotides for the aforementioned reactions could be liberated by the proposed rapid degradation of host RNA [26]. Adele also encodes NAD^+^ reconstitution pathway 1, enabling regeneration of NAD^+^ from nicotinamide and ADP-ribose and thereby counteracting NAD^+^ depletion triggered by abortive infection systems [88]. Although it should be noted, that during the infection by the related PaP1 phage, NAD^+^ concentration significantly decrease even in the presence of these genes [103].

Several predicted regulators of nucleotide-based second messenger systems suggest a deeper interference with host stress responses. A small alarmone hydrolase together with a MazG-like pyrophosphohydrolase may modulate levels of (p)ppGpp, (p)ppApp or related nucleotides produced during nutritional stress and interbacterial competition [104]. In *P. aeruginosa* (p)ppGpp mediate the stringent response, allowing to survive under nutrient limitation and environmental stress and gradually reducing growth and motility [105]. This signaling is complex and probably non-linear, as the loss of small alarmone hydrolase as well as the loss of alarmone production both impair biofilm production [83]. It is intriguing to hypothesize that phage interference in (p)ppGpp signaling leads to metabolic reprogramming and cellular responses ultimately beneficial for phage propagation. Adele also encodes a Dap1 homolog, previously shown to inhibit the c-di-GMP phosphodiesterase DipA and thereby influence motility and virulence in *P. aeruginosa* [87]. This combination together with metabolic reprogramming likely increases robustness of replication across diverse host physiological states.

Adele’s tail fiber genes, metabolic enzymes and anti-defense cassettes exemplify the mosaic nature of phage genomes. N-terminal tail fiber regions responsible for virion attachment are relatively conserved and related to those of distant myoviruses, whereas C-terminal receptor-binding domains are highly diverse and, in some cases, most similar to proteins from phages infecting other bacterial species. This structure is compatible with frequent domain swapping, recombination and occasional host jumps, in which receptor-binding modules circulate among otherwise unrelated backbones. Anti-defense and metabolic genes in Adele are closely related to bacterial proteins and to anti-defense modules previously described in other phages, supporting the view that defense and counter-defense elements form a shared pool of mobile genetic cassettes that are repeatedly captured and re-deployed in the ongoing arms race between phages and bacteria [106].

Structural analysis of Adele’s major capsid protein places it firmly within the HK97-fold lineage, whose reach extends far beyond classical bacteriophages. HK97-like capsids are found not only in *Duplodnaviria* viruses but in viral-like bacterial encapsulin nanocompartments [101,107]. This structural continuum extends to gene transfer agents and other virus-like particles and includes the newly described mirusviruses that bridge tailed phages, herpesviruses and giant viruses [72,108]. The phylogeny based on structural similarity underlines the complex nature of HK97 fold capsids, placing encapsulins in two distant branches and, strikingly, clustering GTAs and cf-PICIs into a distinct common branch. Similarity of capsid proteins of a *Rhodobacter capsulatus* GTA and a cf-PICI was noted earlier [73], but the DALI based phylogeny presented here may suggest a common origin of these, seemingly very different entities, likely involving captured phages that were once fully functional. These observations resonate with the “virus world” and “viral hallmark gene” concepts, which posit that certain structural and informational proteins were subsequently disseminated across diverse viral and virus-like entities [109].

The analyses performed revealed an evolutionary relatedness of *Vandenendeviridae* to several taxa, including the genus *Saclayvirus*, the family *Andersonviridae*, and the subfamilies *Vequintavirinae* and *Stephanstirmvirinae*. This relatedness is reflected in the similarity of hallmark proteins, including the terminase, portal protein and major capsid protein, in the architecture of individual genomic modules and of the genomes as a whole, as well as in virion morphotype and infection cycle. Taken together, these results may be viewed as an argument for assigning the above-mentioned groups to a separate higher-rank taxon (e.g., an order).

The evolutionary analysis of phage Adele, and by extension of bacteriophages more generally, at multiple evolutionary scales underscores the diversity and importance of genetic exchanges between viruses, cellular organisms and virus-like particles. These exchanges not only play a major role in modulating phage host specificity, with direct practical implications, including for phage therapy, but also give rise to new biological entities, thereby shaping not only the virosphere but the evolution of the biosphere as a whole.

## 5. Conclusions

In this work, *Pseudomonas* phage Adele, a lytic myovirus representing a novel species within the genus *Pakpunavirus*, was isolated and comprehensively characterized. Phenotypic assays, biofilm experiments and two larval infection models demonstrated that Adele efficiently infects and lyses non-O11 serogroup *P. aeruginosa*, including clinical isolates, and can provide measurable protection in vivo, which supports its potential as a candidate for phage therapy. Genomic analysis showed that Adele has a compact genome consistent with a strictly lytic infection cycle and lacking identifiable antibiotic resistance, integrase or known toxin genes, but encoding multiple metabolic and anti-defense functions that may facilitate replication in stressed or treated hosts. Comparative genomics, structural modeling and phylogenetic reconstruction revealed a conserved morphogenesis module that includes HK97-fold major capsid proteins, portals, terminases and a triplicated tail tube locus, consistent with predominantly vertical evolution of the head and tail core. At the same time, the organization and sequence diversity of tail fibers and receptor binding proteins, together with evidence for subdomain level exchanges, point to modular and domain specific horizontal gene transfer that likely underlies host range diversification. Overall, Adele combines properties of a promising therapeutic agent with a rich and traceable evolutionary history, making it a useful system for linking structural, genomic and ecological perspectives on phage biology.

## Figures and Tables

**Table 1:** Local virulence values for phage Adele infecting *P. aeruginosa* PAO1.

MOI	Local Virulence
10^−7^	0.17
10^−6^	0.16
10^−5^	0.20
10^−4^	0.25
10^−3^	0.68
0.01	0.97
0.1	0.92
1	0.97

## Data Availability

All relevant data are available within this article and its Supplementary Materials. The *Pseudomonas* phage Adele genome sequence is deposited in NCBI GenBank under accession number PV469300.

## Supplementary Materials

The following supporting information can be downloaded at: https://www.mdpi.com/article/10.3390/v18010042/s1, Table S1: Sensitivity of *Pseudomonas aeruginosa* isolates with sequenced genomes to *Pseudomonas* phage Adele. ST, sequence type determined using PubMLST; ND, no data; non-typeable. Figure S1: Maximum-likelihood phylogenetic trees inferred from 100 representative amino acid sequences of the endonuclease domain of the terminase large subunit, rooted to *Gordonia*-infecting phages (genera *Chidieberevirus* and *Bendigovirus*). Bootstrap support values are shown next to the corresponding nodes; nodes with bootstrap support lower than 50% are collapsed into polytomies. The scale bar indicates the expected number of substitutions per site. Figure S2: Structural gene cluster comparison of *Pseudomonas* phage Adele (family *Vandenendeviridae)* and related phages. Linkers connect best hits obtained using DaliLite.v5 pairwise alignments of AlphaFold3-preditcted structures with Z-scores > 7. Colors represent the functional roles of proteins within the phage particles.

## Abbreviations

The following abbreviations are used in this manuscript:

bp	base pair
cf-PICI	Capsid-forming phage-inducible chromosomal island
CFU	Colony-forming units
GTA	Gene transfer agent
ICTV	International Committee on Taxonomy of Viruses
MCP	Major capsid protein
MOI	Multiplicity of infection
OD	Optical density
PICI	Phage-inducible chromosomal island
PFU	Plaque-forming units
SEM	Scanning electron microscopy
TLS	Terminase large subunit
