# Supplementary figures and images for: Genomic, Evolutionary and Phenotypic Insights into Pseudomonas Phage Adele, a Novel Pakpunavirus with Potential for Phage Therapy
Source: Viruses. 2025 Dec 25;18(1):42. doi: 10.3390/v18010042 (PMC12846349; doi:10.3390/v18010042)

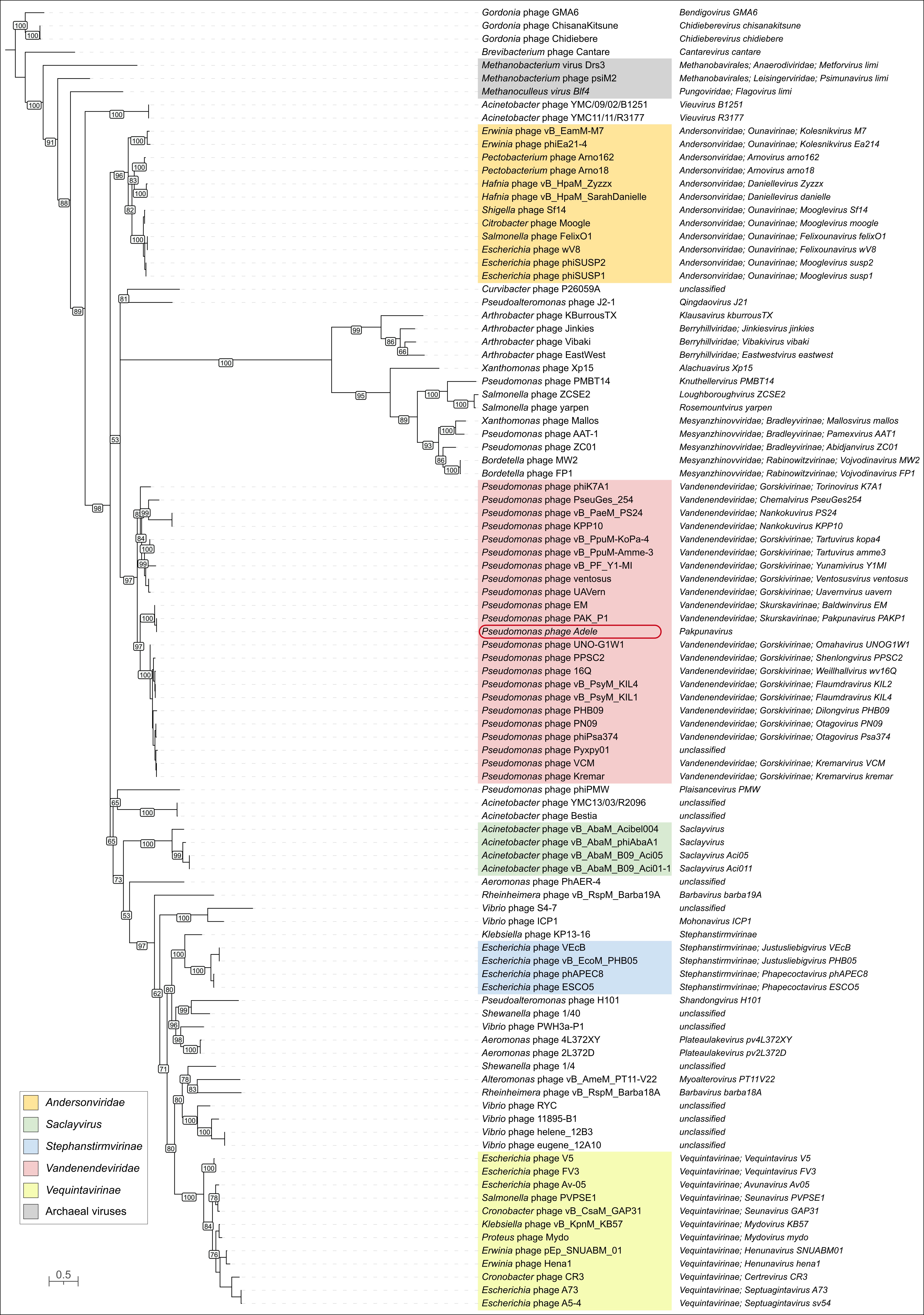

Supplement: Supplementary file 1 [file viruses-18-00042-s001.zip › viruses-4029695-supplementary/Supplementary_Figure_S1.jpg]

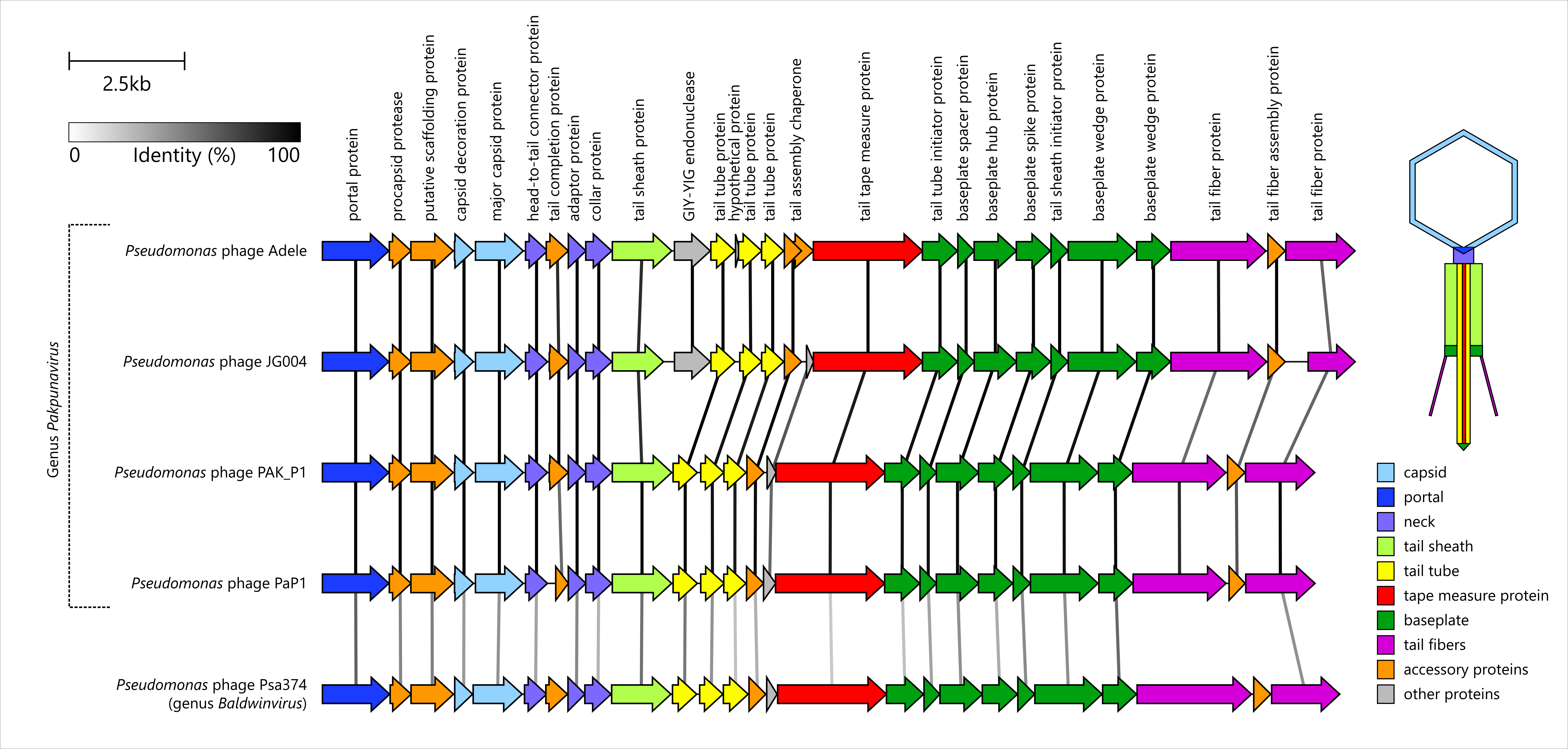

Supplement: Supplementary file 1 [file viruses-18-00042-s001.zip › viruses-4029695-supplementary/Supplementary_Figure_S2.png]
